# NSD2 contributes to oncogenic RAS-driven transcription in lung cancer cells through long-range epigenetic activation

**DOI:** 10.1038/srep32952

**Published:** 2016-09-08

**Authors:** Verónica García-Carpizo, Jacinto Sarmentero, Bomie Han, Osvaldo Graña, Sergio Ruiz-Llorente, David G. Pisano, Manuel Serrano, Harold B. Brooks, Robert M. Campbell, Maria J. Barrero

**Affiliations:** 1 CNIO-Lilly Epigenetics Laboratory, Spanish National Cancer Research Center (CNIO), C/ Melchor Fernández Almagro, 3. 28029 Madrid, Spain; 2 Eli Lilly and Company, 46285 Indianapolis, USA; 3 Structural Biology and Biocomputing Program, Spanish National Cancer Research Center (CNIO), C/ Melchor Fernández Almagro, 3. 28029 Madrid, Spain; 4 Molecular Oncology Program, Spanish National Cancer Research Center (CNIO), C/Melchor Fernández Almagro, 3. 28029 Madrid, Spain

## Abstract

The histone methyltransferase NSD2/WHSC1/MMSET is overexpressed in a number of solid tumors but its contribution to the biology of these tumors is not well understood. Here, we describe that NSD2 contributes to the proliferation of a subset of lung cancer cell lines by supporting oncogenic RAS transcriptional responses. NSD2 knock down combined with MEK or BRD4 inhibitors causes co-operative inhibitory responses on cell growth. However, while MEK and BRD4 inhibitors converge in the downregulation of genes associated with cancer-acquired super-enhancers, NSD2 inhibition affects the expression of clusters of genes embedded in megabase-scale regions marked with H3K36me2 and that contribute to the RAS transcription program. Thus, combinatorial therapies using MEK or BRD4 inhibitors together with NSD2 inhibition are likely to be needed to ensure a more comprehensive inhibition of oncogenic RAS-driven transcription programs in lung cancers with NSD2 overexpression.

NSD2 (nuclear receptor-binding SET domain-containing 2), also known as MMSET (multiple myeloma SET domain) or WHSC1 (Wolf-Hirschhorn syndrome candidate 1) is a histone methyltransferase that belongs to the NSD family of SET domain-containing methyltransferases which also includes NSD1 and NSD3. Deletions in NSD2 cause the Wolf-Hirschhorn syndrome (WHS) characterized by delayed growth and intellectual disability while NSD2 overexpression has been linked to cancer (reviewed in Morishita and Di Luccio^1^).

NSD2 shows gain of function in blood cancers due to fusions to the IgH locus via t(4;14) translocations that cause its overexpression in multiple myeloma^2^,^3^ or recurrent E1099K mutations that enhance its methyltransferase activity in lymphomas^4^,^5^,^6^. Additionally, NSD2 has been reported to be upregulated in a number of solid cancers such as squamous cell carcinoma of the head and neck^7^, endometrial cancer^8^, lung cancer^9^,^10^, neuroblastoma^11^, bladder and colon cancer^9^,^10^, hepatocellular carcinoma^12^, ovarian carcinoma^13^ and prostate cancer^14^. Overexpression in solid tumors appears to occur in the absence of genetic alterations. Additionally, NSD2 has been demonstrated to support the proliferation and/or survival of several cancer cell lines including myeloma cell lines with t(4;14) translocations^15^,^16^,^17^,^18^, leukemia cell lines carrying the E1099K mutation^4^, prostate cancer^14^,^19^,^20^ and osteo and fibrosarcoma cell lines^15^.

The role of NSD2 has been linked to transcriptional elongation through interactions with BRD4, pTEFb and HIRA^21^,^22^,^23^. Two independent studies have suggested that BRD4 can mediate the recruitment of NSD2 to the transcription start sites (TSS) of certain genes^21^,^22^. Interactions of NSD2 with BRD4 and pTEFb at the TSS are likely to play roles in RNA Pol II pause release while interactions with HIRA facilitate H3.3 deposition during elongation towards the transcribed end of genes^22^. NSD2 mediates mono and dimethylation of H3K36^15^,^18^. Although the precise role of H3K36me1/2 in transcriptional activation is unclear, it has been suggested that it might serve as a substrate for SETD2, a histone methyltransferase involved in elongation that is not able to mono and dimethylate H3K36^24^ and likely uses the substrate modified by NSD2 to achieve H3K36 trimethylation on coding regions^25^.

Despite the fact that NSD2 has been reported to be frequently overexpressed in lung cancer, the contribution of NSD2 to the malignancy of this disease is poorly understood. Here, we describe that NSD2 contributes to the proliferation of a subset of lung cancer cell lines by altering oncogenic RAS transcriptional responses. Combinatorial therapies using MEK inhibitors or BRD4 inhibitors together with NSD2 inhibition are likely to be effective in fighting RAS-dependent cancers with NSD2 overexpression.

## Results

### NSD2 is highly expressed in a subset of lung cancer cell lines

**Figure 1:**
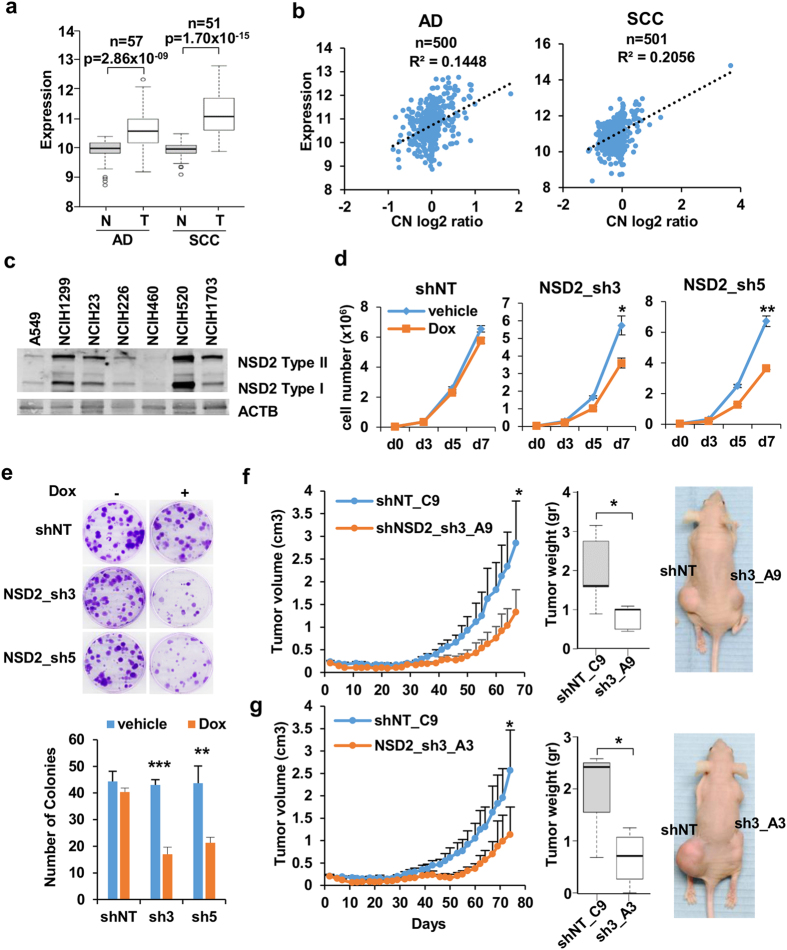
NSD2 is overexpressed in lung cancer and contributes to support the growth of lung cancer cell line H1299. (**a**) Box plot of mRNA levels of NSD2 in paired normal (N) and tumor (T) tissues analyzed by RNA-seq by The Cancer Genome Atlas (TCGA) in lung adenocarcinoma (AD) and squamous cell carcinoma (SCC) patients. P-values were calculated using paired *t*-test. (**b**) Correlation between copy number (CN) and expression levels in lung cancer samples available from TCGA. (**c**) Levels of NSD2 determined by western blot in selected lung cancer cell lines. Figure S1 shows mRNA expression and copy number in lung cancer cell lines. (**d**) Growth curves of H1299 transduced with doxycycline inducible non target shRNA (shNT) and two different shRNAs against NSD2 (sh3 and sh5) in the presence of vehicle or doxycycline. Graph shows mean and standard deviation of triplicates from one representative experiment out of five. *p-value < 0.05 and **p-value < 0.005 determined by *t-*test. Figure S1 shows validation and location of six different shRNAs. (**e**) Colony forming assays of cell lines used in D in the presence of vehicle (−) or doxycycline (+). Left panel shows crystal violet staining of colonies. Graph shows mean and standard deviation of colony count in three replicates from one representative experiment out of three. **p-value < 0.005 and ***p-value < 0.0005 determined by *t-*test. (**f** ) Xenograft in doxycycline treated nude mice injected with one non target clone of H1299 (shNT_C9) and one NSD2 sh3 clone (NSD2_sh3_A9) in each flank. Left panel shows tumor volume measured at the indicated days after injection and central panel shows tumor weight at the time of sacrifice. Right panel shows one representative animal. Mean and standard deviation from five animals is shown. *p-value < 0.05 determined by paired *t-*test. Supplementary Fig. 4 shows the characterization of the selected clones. (**g**) As in F but comparing one non target clone of H1299 (shNT_C9) and a different NSD2 sh3 clone (NSD2_sh3_A3). Mean and standard deviation from four animals is shown. *p-value < 0.05 determined by paired *t-*test.

To confirm previous reports on NSD2 overexpression in lung cancer^9^,^10^ we analyzed data from The Cancer Genome Atlas (TCGA). Analysis of mRNA levels showed that NSD2 is significantly overexpressed in lung adenocarcinoma (AD) and squamous cell carcinoma (SCC) when compared with normal lung tissue obtained from the same patients (Fig. 1a). Evaluation of the differential expression of 23 additional histone lysine methyltransferases between normal and lung tumor tissues showed that NSD2 is among the most significantly upregulated histone methyltransferases both in AD and SCC compared to normal tissues (Supplementary Fig. 1a,b). As previously reported, high expression of NSD2 in lung tumors did not significantly correlate with copy number gain (Fig. 1b).

To verify whether lung cancer cell lines could recapitulate the same phenomena as lung tumors we screened the expression of NSD2 by western blot in a panel of cell lines that were available in our laboratory (Fig. 1c). Several cell lines expressed detectable levels of NSD2 protein while in others levels were very low or almost undetectable. Examination of data from the Cancer Cell Line Encyclopedia (CCLE) revealed good correlations between protein expression and mRNA levels in most tested cell lines but no significant correlation with copy number (Supplementary Fig. 2a), suggesting that NSD2 overexpression cannot be explained by copy number gain in these cell lines.

### NSD2 knock down causes growth defects in H1299

We next evaluated potential consequences of NSD2 knock down in the lung cancer cell line H1299 that expresses high levels of NSD2. Cells were infected with lentiviruses generated with the pTRIPZ lentiviral vector back bone to drive the expression of six different shRNAS targeting NSD2 at different locations (Supplementary Fig. 2b). Infected cells were treated with puromycin to generate resistant stable cell lines in which the shRNA expression is induced in the presence of doxycycline. After 6 days of doxycycline treatment, the expression of NSD2 was interrogated by western blot using an antibody raised against the N-terminal domain of NSD2 that allowed the detection of previously described isoforms type I and II (Supplementary Fig. 2c). Short hairpin sh3 was very efficient in depleting both isoforms, while sh5 and sh6 had milder but significant efficiency over both isoforms. As expected from its localization sh4 targeted only the type II isoform (Supplementary Fig. 2c). Since the antibody used was raised against the N-terminal domain, it did not allow the detection of the previously described RE-IIBP isoform which is translated from a mRNA with a different transcription initiation^26^.

Cell lines transduced with a non-target shRNA (shNT), sh3 and sh5 were evaluated for potential effects on proliferation. Proliferation curves (Fig. 1d) showed that in the presence of doxycycline cell lines transduced with sh3 and sh5 have a lower cell count compared to non-treated cells. Importantly, doxycycline treatment had no significant effects in shNT cells. Colony formation assays revealed about 50% less colonies in sh3 and sh5-transduced cells treated with doxycycline for 15 days (Fig. 1e). To further validate our shRNAs, we transduced leukemia and lymphoma cell lines with NSD2-gain of function reported to be dependent on NSD2 expression for proliferation^4^,^18^. Both cell lines, KMS11, a multiple myeloma line which harbors the t(4;14) translocation, and RCH-ACV, a lymphoblastic leukemia line with the E1099K activating mutation, were sensitive to NSD2 depletion mediated by sh3 and sh5 (Supplementary Fig. 2d,e).

To interrogate the specificity of the NSD2 depletion effects over other members of the NSD family of H3K36 methyltransferases we knocked down the expression of NSD3 in H1299. Knock down of NSD3 did not have significant effects in the proliferation of H1299 cells, neither alone or in combination with the NSD2 knock down (Supplementary Fig. 3).

Last, we evaluated the ability of NSD2 to support H1299 cell growth *in vivo* in a mouse xenograft model system. To ensure maximum stability of the NSD2 knock down in the absence of puromycin, we selected clones from sh3 or shNT-infected cells with high levels of shRNA expression (see Supplemental Experimental Procedures). As expected, these clonal cell lines showed good NSD2 knock down (Supplementary Fig. 4a) and more significant effects on proliferation upon doxycycline treatment (Supplementary Fig. 4b,c) than pooled cell lines (Fig. 1d). Importantly, effects in proliferation correlated with an increase in the number of cells in G0/1 after NSD2 depletion (Supplementary Fig. 4d). Nude mice were injected subcutaneously with one shNT and two different sh3 clones, treated with doxycycline and tumors were monitored for about two and a half months. Both sh3 clonal cell lines showed lower tumor volume along time and lower tumor weight at the time of sacrifice than the shNT clonal cell line (Fig. 1f,g).

### NSD2 knock down reduces the global levels of H3K36me2

**Figure 2:**
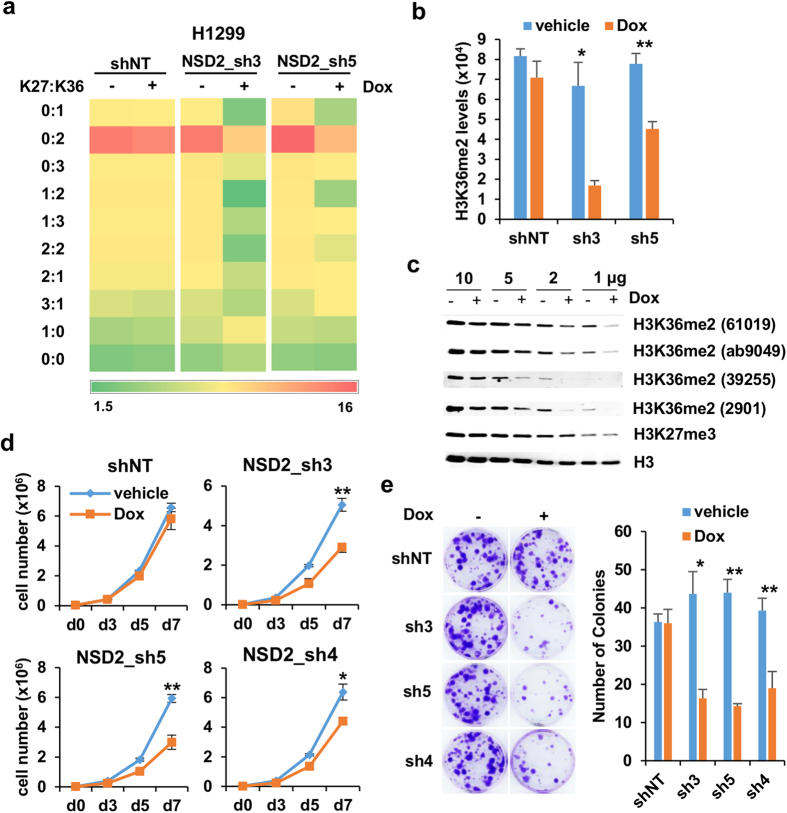
Changes in H3K36 methylation in H1299 after NSD2 knock down. (**a**) Heat map of the abundance of histone H3 K27 and K36 methylation as determined by mass spectrometry in transduced H1299 cell lines in the presence of vehicle (−) or doxycycline (+). Co-presence of non-methylated (0), monomethylated (1), dimethylated (2) and trimethylated (3) at the indicated residues is shown. Abundance is uncalibrated signal intensity of the indicated peptide as a percentage among all peptides that contained K27 and K36. Two of the most abundant species, K27:K36_2:0 and_3:0 are not shown. (**b**) Levels of H3K36me2 were determined in the same samples as in A using AlphaLisa. Mean and standard deviation from triplicates is shown. *p-value < 0.05 and **p-value < 0.005 determined by *t-*test. (**c**) Levels of H3K36me2 were determined in H1299 cells transduced with sh3 and treated with vehicle (−) or doxycycline (+) using different antibodies against H3K36me2 (61019 and 39255 from Active Motif, ab9049 from Abcam and 2901 from Cell Signaling) and loading decreasing amounts of total cell extract. Total H3 content was used as a loading control. (**d**) Growth curves of H1299 transduced with doxycycline inducible non target shRNA (shNT) and two different shRNAs that target both type I and type II NSD2 (sh3 and sh5) and a shRNA that targets NSD2 type II only (sh4) in the presence of vehicle or doxycycline. Graph shows mean and standard deviation of three replicates. *p-value < 0.05 and **p-value < 0.005 determined by *t-*test. (**e**) Colony forming assays of cell lines used in D in the presence of vehicle (−) or doxycycline (+). Upper panel shows crystal violet staining of colonies. Graph shows mean and standard deviation of colony count in three replicates from one representative experiment out of two. *p-value < 0.05 and **p-value<0.005 determined by t-test.

Depletion of NSD2 in myeloma, leukemia and prostate cancer cell lines reduces the global levels of H3K36me2^4^,^15^,^18^,^20^. To verify whether the knock down of NSD2 in the lung cancer cell line H1299 causes similar changes in histone methylation we conducted mass spectrometry analysis to monitor the status of histone H3 K36 methylation in combination with K27 methylation that can be readily detected on the same peptide (Fig. 2a). Among the peptides that contain methylation on H3K36, the most abundant species corresponded to dimethylated K36 combined with non-modified K27. This was also the most conspicuously downregulated combination after NSD2 knock down. Co-occurrence of methylation of both K36 and K27 in the same peptide had low frequency as expected from the reported opposite roles in transcription regulation of both marks^27^. Importantly, the global changes in H3K36me2 observed by mass spectrometry could be reproduced when using an AlphaLISA system (Perking Elmer) to detect H3K36me2 (Fig. 2b). Last, four different commercial antibodies raised against H3K36me2 tested by western blot showed consistent reductions in the levels of this mark upon doxycycline treatment (Fig. 2c). Unlike reported in myeloma cell lines with t (4;14) translocations^18^, we did not detect global changes in H3K27me3 after NSD2 depletion by western blot (Fig. 2c), however we cannot rule out that local changes in this mark are taking place.

Previous work demonstrated that the methyltransferase activity of NSD2 is required to mediate the effects on proliferation in myeloma cells^15^. To gain insight into the involvement of the methyltransferase activity in the proliferation of lung cancer cells we tested the effects on proliferation of shRNA sh4 which specifically targets the NSD2 type II isoform that contains the SET domain (Supplementary Fig. 2c). Figure 2d,e show that the knock down of the type II isoform causes a decrease in proliferation, suggesting that the methyltransferase activity is important to support the growth of H1299 cells.

### The NSD2 knock down interferes with the RAS-transcriptional program

**Figure 3:**
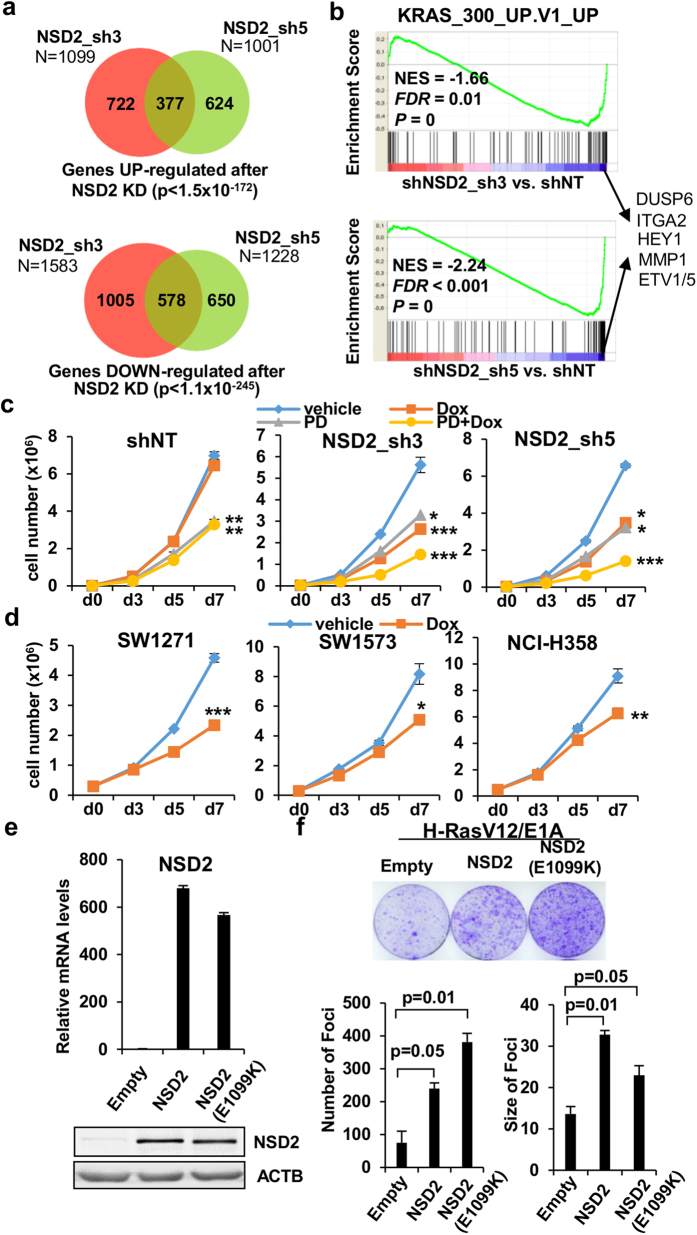
NSD2 supports the RAS oncogenic program. (**a**) Overlap of genes significantly upregulated (upper panel) or downregulated (lower panel) by NSD2 shRNAs sh3 and sh5 at a FDR < 0.05. P-values for the significance of the overlap of genes affected by the two shRNAs is indicated. (**b**) Gene set enrichment analysis plot showing significant enrichment of RAS-associated gene signature (using KRAS_300_UP.V1_UP gene set from the Molecular Signature Database) in genes downregulated by the sh3 (upper panel) and sh5 (lower panel). Several genes common to the leading edge of both shRNAs are shown. (**c**) Growth curves of H1299 transduced with doxycycline inducible non target shRNA (shNT) and two different shRNAs against NSD2 (sh3 and sh5) in the presence of vehicle (DMSO), doxycycline (hereafter labelled as Dox) and/or 10 nM MEK inhibitor PD0325901 (hereafter labelled as PD). Graph shows mean and standard deviation of three replicates from one representative experiment out of two. *p-value < 0.05 and ***p-value < 0.0005 determined by *t*-test comparing each treatment to vehicle. (**d**) Growth curves of SW1271, SW1573 and NCI-H358 cells transduced with sh3 in the presence of vehicle and doxycycline. *p-value < 0.05, **p-value < 0.005 and ***p-value < 0.0005 by t-test. (**e**) Levels of NSD2 mRNA (upper panel) and protein (lower panel) in MEFs infected with empty vector (empty), pMSCV-FLAG-NSD2-Puro (NSD2) or gain of function mutant NSD2 (E1099K) (**f**) Upper panel shows crystal violet staining of RAS- transformed MEFs infected with empty (empty), wild type NSD2 (NSD2) or mutant NSD2 overexpressing retroviruses (NSD2 E1099K). Lower panel shows the mean and standard deviation of the number and size of foci for each condition. P-values according to t-test are also shown.

We hypothesized that the dramatic changes in H3K36me2 content after NSD2 depletion would lead to important changes in gene expression. To test this, we assessed genome-wide gene expression by RNA-seq in H1299 cells infected with shNT, sh3 or sh5 and treated with doxycycline. Differentially upregulated and downregulated genes comparing sh3 or sh5 to shNT where selected at FDR < 0.05 (Fig. 3a). Since NSD2 is involved in transcriptional activation, genes downregulated after NSD2 knock down are more likely to be NSD2 targets. Gene set Enrichment Analysis (GSEA)^28^ revealed that the RAS pathway was at the top of most significantly downregulated pathways. Downregulated genes were enriched in genes that are upregulated when KRAS is overexpressed in epithelial cancer cells (MSigDB signature KRAS_300_UP.V1_UP; Fig. 3b). The H1299 line harbors an activating mutation in NRAS and has been previously described to be sensitive to MEK inhibitors^29^. Co-treatment of H1299 cell lines with doxycycline and the MEK inhibitor PD0325901^30^, resulted in additive effects on proliferation (Fig. 3c) suggesting that both treatments might cooperate in the RAS pathway to affect cell growth. Moreover, depletion of NSD2 in other lung cancer cell lines with RAS activating mutations and high levels of NSD2 (Fig. 3d) had antiproliferative effects, further confirming the impact of NSD2 on the transcriptional responses of oncogenic RAS in lung cancer cell lines. Knock down of NSD2 in cell lines H520 and H1703 that express high levels of NSD2 but do not have RAS mutations did not have effects in proliferation (Supplementary Fig. 5a–d). Finally, we confirmed that NSD2 facilitates E1A-Ras-mediated transformation of mouse embryonic fibroblasts. Overexpression of NSD2 increased the number and more significantly the size of E1A-Ras-transformed foci (Fig. 3e,f). Importantly, overexpression of the catalytic mutant (E1099K) with enhanced methyltransferase activity^4^,^5^,^6^ further increased the number of foci (Fig. 3f), suggesting that the methyltransferase activity on NSD2 is involved in supporting transformation.

### NSD2 depletion and JQ1 treatment cooperate to prevent lung cancer cell growth

Members of the NSD family, including NSD2, have been described to interact with the bromodomain containing protein BRD4 which might participate in their recruitment to chromatin^21^,^22^,^23^. BRD4 is recruited through its bromodomain to regions of the genome with high levels of histone acetylation that have been called super-enhancers^31^. Inhibitors that block the interaction of BRD4 with acetylated histones have been demonstrated to have antiproliferative effects due to the silencing of oncogenes that are dependent on super-enhancers to maintain their high levels of expression in cancer cells^32^. Additionally, the recent finding that BRD4 supports oncogenic RAS-driven transcription in malignant peripheral nerve sheath tumors^33^ prompted us to check potential cooperation between NSD2 depletion and the treatment with the BRD4 inhibitor JQ1. Supplementary Fig. 5e shows that JQ1 has antiproliferative effects in H1299 cells and that combination of doxycycline and JQ1 treatment has additive effects on proliferation.

### PD0325901, JQ1 and NSD2 knock down cooperate on the RAS pathway through commonly and uniquely regulated genes

**Figure 4:**
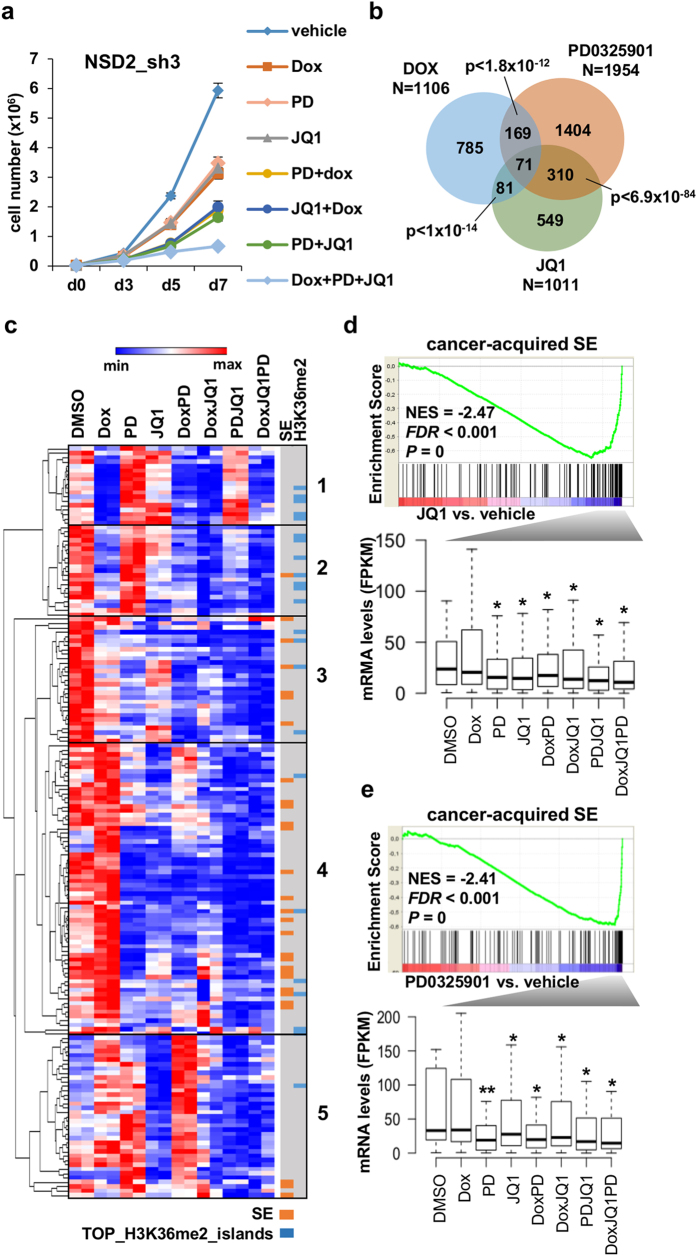
Cooperative responses of NSD2 knock down, PD0325901 and JQ1 treatments. (**a**) Growth curves of H1299 transduced with doxycycline inducible NSD2 shRNA sh3 in the presence of vehicle (DMSO), doxycycline, 10 nM PD0325901 and/or 75 nM JQ1. Graph shows mean and standard deviation of three replicates from one representative experiment out of two. All treatments had significant effects compared to the vehicle at a p-value < 0.0005 except JQ1 treatment at a p-value < 0.005 as determined by *t*-test. (**b**) Overlap of genes significantly downregulated (FDR < 0.05) by the indicated independent treatments compared to the vehicle (DMSO). The total number of downregulated genes by each treatment is also shown. P-values for the significance of the overlap of downregulated genes between two treatments are shown. (**c**) Clustered heat map of the expression of genes contributing the most to the RAS signature and significantly downregulated (FDR < 0.05) by the independent treatments. Association with super-enhancers and overlap with top H3K36me2 methylated islands is indicated. Main clusters of genes with similar patterns of gene expression are indicated. (**d**) Upper panel shows gene set enrichment analysis (GSEA) plot showing significant enrichment of cancer-acquired super-enhancers-associated gene signature and genes downregulated by JQ1. Lower panel shows the expression of genes on the leading edge for each treatment. *p-value < 0.05 determined by paired t-test comparing each treatment to the vehicle (DMSO). (**e**) Upper panel shows gene set enrichment analysis (GSEA) plot showing significant enrichment of cancer-acquired super-enhancers-associated gene signature and genes downregulated by PD0325901. The expression of genes on the leading edge is shown in the lower panel. *p-value < 0.05, **p-value < 0.005 determined by paired t-test comparing each treatment to the vehicle (DMSO).

To understand how PD0325901, JQ1 and NSD2 depletion cooperate to prevent the growth of lung cancer cells we analyzed by RNA-seq the changes in gene expression caused by the individual treatments and in combination (Fig. 4a). PD0325901 caused the downregulation of the largest number of genes and showed a larger overlap with JQ1 than with doxycycline treatment (Fig. 4b; see Supplementary Table 1 for a list of downregulated genes).

Despite the reported ability of JQ1 to interfere with MYC expression and MYC-driven transcription^32^,^34^ JQ1 did not significantly affect the expression of *MYC* nor downregulated the MYC transcriptional program (Supplementary Fig. 6a). Only the treatment with PD0325901 caused a significant downregulation of *MYC* expression that was lost after combination with the other treatments. On the contrary, GSEA revealed that all three individual treatments caused downregulation of the expression of genes upregulated by the RAS pathway but each treatment showed maximum enrichment in different RAS-dependent gene sets (Supplementary Table 2), suggesting that each treatment could be affecting the expression of a different subset of RAS target genes. To better understand the co-operation of the different treatments on these gene sets we compiled a list of genes from the RAS signatures shown in Supplementary Table 2 significantly downregulated by the individual treatments (Supplementary Table 3) and compared their expression between treatments. Supplementary Fig. 6b shows partial overlap on downregulated genes of the RAS pathway between treatments and cooperative downregulation on commonly targeted genes in combined treatments (Supplementary Fig. 6c). Clustering of the RAS-signature genes according to changes in expression shows five main clusters of commonly behaving genes (Fig. 4c). Clusters 1 to 3 contain genes downregulated by NSD2 knock down but only genes in cluster 3 are also downregulated by JQ1 and PD0325901 treatment. Cluster 4 contains the largest number of genes which are targeted by JQ1 and PD0325901 but not NSD2. Overall our analysis suggests that all three treatments converge on the RAS pathway by affecting the expression of common cooperatively targeted genes and uniquely targeted genes. JQ1 and PD0325901 show the largest overlap on the RAS signature, while NSD2 depletion seems to affect a different subset of RAS-target genes. In general, genes that are downregulated by one single treatment remain downregulated in the triple treatment (Fig. 4c).

Importantly, phospho-ERK was unaffected by NSD2 depletion or by JQ1 treatment (Supplementary Fig. 6d) suggesting that BRD4 and NSD2 affect the RAS pathway by directly modulating the RAS transcriptional output at the level of chromatin.

### PD0325901 and JQ1 downregulate the expression of genes associated with cancer-acquired super-enhancers

JQ1 causes downregulation of oncogenes regulated by super-enhancers, affecting the proliferation of cancer cells^32^. Therefore, we speculated that the cooperation between JQ1 treatment and NSD2 knock down might be through crosstalk between H3K36me2 and histone acetylation. In order to address this question, we identified super-enhancers in H1299 and in normal lung according to H3K27ac density signal (Supplementary Fig. 7a) using the software ROSE (Rank Ordering of Super-Enhancers) as previously described^32^,^35^. Comparison of the density of H3K27ac at super-enhancer regions between both samples shows that some super-enhancers are present in both H1299 and normal lung, but others are heavily H3K27 acetylated only in H1299 and are likely to be specifically acquired during oncogenic transformation (Supplementary Fig. 7b; Supplementary Table 3). Moreover, some of these cancer-acquired super-enhancers are associated with genes upregulated in lung cancer samples compared to normal tissues (Supplementary Fig. 7c). Additionally, genes associated with cancer-acquired super-enhancers in H1299 were found to be significantly enriched in genes upregulated by the KRAS pathway (MSigDB Oncogenic Signature “Genes up-regulated in epithelial lung cancer cell lines over-expressing an oncogenic form of KRAS gene” p-value = 12.63 × 10^−07^ determined by GREAT)^36^.

Gene set enrichment analysis shows that genes downregulated by JQ1 treatment were enriched in genes associated with cancer-acquired super-enhancers (Fig. 4d upper panel) but not significantly enriched in genes associated with super-enhancers shared between H1299 and normal lung at a FDR < 0.05 (Supplementary Table 2). Genes downregulated by JQ1 contributing the most to the cancer-acquired super-enhancer signature (leading-edge genes) were also significantly downregulated by PD0325901 but not by doxycycline (Fig. 4d lower panel). In accordance PD0325901 treatment contributed to downregulate genes associated with cancer-acquired super-enhancers (Fig. 4e) and less significantly to those shared between both samples (Supplementary Table 2) and genes downregulated by PD0325901 contributing the most to the cancer-acquired super-enhancer signature were significantly downregulated by JQ1 (Fig. 4e lower panel). These results suggest that the RAS pathway through activation of MEK has a critical role in establishing and/or maintaining the functionality of cancer-acquired super-enhancers in H1299.

### NSD2 regulates the expression of genes marked with H3K36me2

Although previous reports described interactions between BRD4 and NSD2, there was a lack or enrichment of the cancer-acquired super-enhancer signature in genes changing expression after the NSD2 knock down (Supplementary Table 2), and genes contributing the most to the cancer-acquired super-enhancer signature in H1299 cells treated with JQ1 were not significantly changing after NSD2 knock down (Fig. 4d lower panel). These results suggest that in H1299, NSD2 is supporting the RAS-driven transcriptional pathway independently of BRD4. To ascertain which genes are direct targets of NSD2, we conducted ChIP-seq of H3K36me2 in H1299 cells transduced with sh3 in the presence and absence of doxycycline.

The general profile of H3K36me2 in vehicle treated H1299 cells recapitulated basic features previously described for this mark, including positive correlation with marks of active transcription and negative correlation with the repressive H3K27me3 mark^18^,^27^ (Supplementary Fig. 8a). Analysis of H3K36me2 enrichment in 5Kb around the TSS of genes showed that this mark is enriched in genes that are expressed versus those that are not expressed (Supplementary Fig. 8b) further confirming that this mark is associated with gene expression. Additionally, we found that H3K36me2 is depleted at CpG islands (Supplementary Fig. 8c). This depletion has been attributed to the recruitment of the H3K36 demethylase KDM2A to these sites^25^. Presence of CpG islands and nucleosome clearance might be responsible for the deep depletion of H3K36me2 detected at the very TSS of actively transcribed genes (Supplementary Fig. 8b). Finally, we confirmed that H3K36me2 and H3K36me3 signals have different profiles^25^,^37^. H3K36me3 is correlated with elongation and increases along the coding region of genes while H3K36me2 is mainly enriched at the TSS and less abundant at coding regions (Supplementary Fig. 8d). Lower levels of H3K36me2 at coding regions are clearly observed at genes with high levels of H3K36me3 (Supplementary Fig. 9a–d). This profile was validated at the *HMGA2* and *MYC* genes by ChIP coupled to qPCR using two different antibodies against H3K36me2 (Supplementary Fig. 9b,d) and it is in agreement with the reported conversion of H3K36me2 into H3K36me3 by SETD2 in coding regions^24^,^25^.

**Figure 5:**
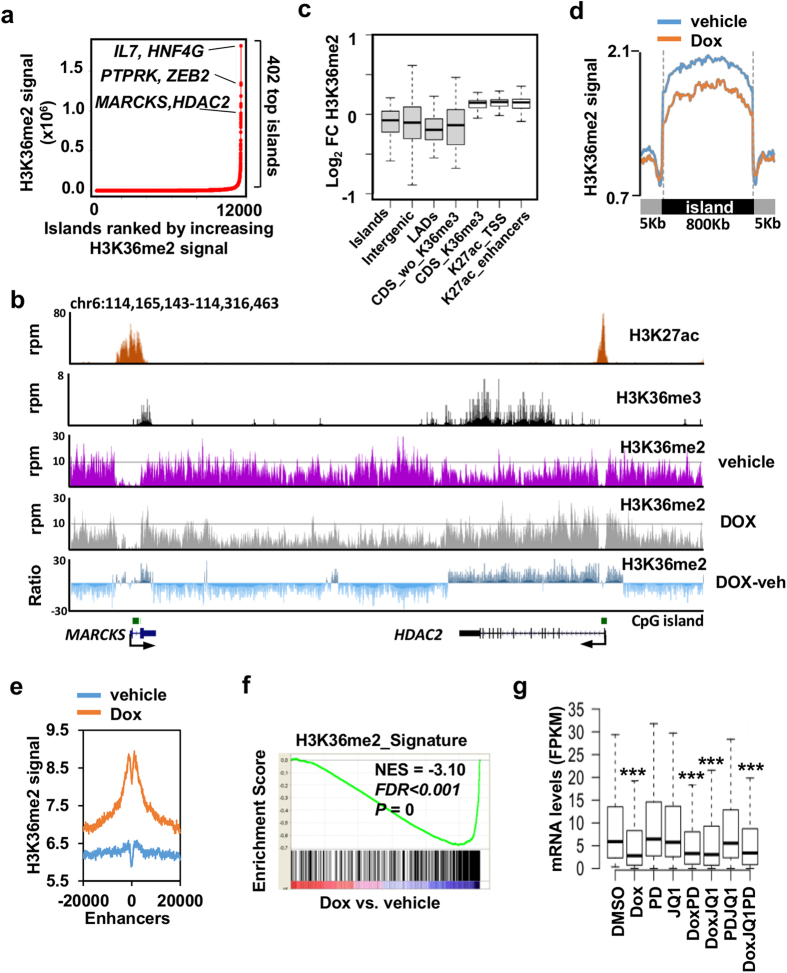
Loss of H3K36me2 correlates with downregulated genes after NSD2 knock down. (**a**) Top H3K36me2 islands in vehicle treated cells identified using ROSE. (**b**) Gene tracks of H3K36me2 signal in presence (dox) or absence (vehicle) of doxycycline. Ratio of H3K36me2 signal in doxycycline minus vehicle treated cells is also shown (Negative light blue values represent loss of signal caused by doxycycline, positive dark blue values represent remaining signal after doxycycline treatment). H3K36me3, H3K27ac signal and CpG islands are also shown. (**c**) Log_2_ of fold change (doxycycline vs. vehicle) of H3K36me2 signal at different genomic features located in top H3K36me2 islands. Significance to the fold change was tested using paired two-tailed *t* test; *P* = 8.63 × 10^−15^ in top H3K36me2 islands (islands), *P *= 5.68 × 10^−34^ for intergenic regions (intergenic) *P* = 1.76 × 10^−19^ for lamina associated domains (LADs), *P* = 1.44 × 10^−15^ for coding regions without H3K36me3 (CDS_wo_K36me3), *P* = 6.531 × 10^−101^ for regions marked with H3K27ac located at TSSs (K27ac_TSS), *P* = 1.70 × 10^−155^ for H3K27ac marked regions not coincident with TSSs (K27ac_enhancers) and *P* = 4.81 × 10^−09^ for coding regions with H3K36me3 (CDS_K36me3). (**d**) Metagene representation of average H3K36me2 density at top H3K36me2 identified in A in the presence of vehicle or doxycycline. The x axis shows the average size of the top H3K36me2 islands flanked by 5 Kb up and down. See Supplementary Table 4 for comparison of H3K36me2 densities at each island in the presence of vehicle or doxycycline. (**e**) Average density of H3K36me2 −/+20 Kbs around the center of H3K27 acetylated regions not coincident with TSSs (enhancers) in top H3K36me2 islands in H1299 cells transduced with sh3 and treated with doxycycline (Dox) or vehicle. (**f** ) Gene set enrichment analysis (GSEA) plot showing significant enrichment of top H3K36me2 islands downregulated by dox-associated gene signature (H3K36me2_Signature; Supplementary Table 3) and genes downregulated by doxycycline. (**g**) Expression of genes on the leading edge of panel F after different treatments. ***p-value < 0.0005 determined by paired t-test comparing each treatment to control (DMSO).

Similar to the analysis done to identify regions with top levels of H3K27ac, we used ROSE to rank the H3K36me2 density signal in vehicle treated cells (Fig. 5a). This analysis revealed that certain genomic locations display very high levels of H3K36me2. After stitching together H3K36me2 intervals separated less than 12.5 Kb, 402 top H3K36me2 regions were identified with an average size of 800 Kb and a maximum size of 5 Mb (Supplementary Table 4). Therefore, in the presence of high levels of NSD2 H3K36me2 regions extended long domains (that we called H3K36me2 islands) that typically embedded several genes, including well known clusters of genes such as the histone cluster (38 genes covered by a single island) or the metalloproteinase cluster. This broad distribution might be expected from an abundant modification described to mark up to 40% of histone H3 in mammalian cells^38^.

We next focused on the changes in H3K36me2 caused by the NSD2 knock down. To have a broad interpretation of these changes along the genome we subtracted the levels of H3K36me2 in vehicle treated cells to the levels found in doxycycline treated cells and displayed the subtraction in the UCSC genome browser. Figure 5b shows a representative genomic region with two genes located in the center of a top H3K36me2 island. Subtraction of signals clearly shows that in doxycycline treated cells H3K36me2 is decreased at intergenic regions while signal is retained at coding regions and proximal promoters, coincident with the presence of H3K36me3 and H3K27ac, respectively. To confirm that this trend is conserved along all top H3K36me2 islands we compared the changes of H3K36me2 at specific genomic locations within the islands (Fig. 5c). We first confirmed that after NSD2 depletion H3K36me2 signal was generally decreased at top H3K36me2 islands (Fig. 5c,d), which is in agreement with the global changes detected by mass spectrometry. Analysis of particular genomic features at top H3K36me2 islands confirmed decrease of H3K36me2 at intergenic locations after NSD2 knock down while signal was retained at coding regions marked with H3K36me3 and regions marked with H3K27ac, especially at enhancer regions (Fig. 5c,e, Supplementary Fig. 8a). This pattern was confirmed by ChIP coupled to qPCR at intergenic regions downstream of the *HDAC2* gene and at the *MYC* super-enhancer (Supplementary Fig. 9e). Additionally, top H3K36me2 islands covered several lamina associated domains (LADs) that lose H3K36me2 methylation after NSD2 depletion (Fig. 5c). Therefore, we conclude that when levels of NSD2 are low H3K36me2 is confined mainly to H3K27ac regions and also the coding regions of genes that are actively transcribed. In the presence of high levels of NSD2 H3K36me2 spreads into intergenic regions including lamina associated domains. In accordance with our results, spreading of the H3K36me2 signal into intergenic regions has been also reported in KMS11 cells^15^.

In order to identify most likely NSD2 target genes, we selected top H3K36me2 islands that loose this signal after NSD2 knock down (Supplementary Table 4) and located the genes whose promoters (−/+ 5Kb around the TSS) are embedded in these islands to build a H3K36me2 signature for GSEA. Strong enrichment of this signature was confirmed in doxycycline-downregulated genes (Fig. 5f), while JQ1 and PD0325901 single and combined treatments did not show significant enrichment (Supplementary Table 2). Consistently, the expression of genes in the leading edge of the H3K36me2 signature was not significantly downregulated by JQ1 or PD0325901 but remained downregulated in combined treatments with doxycycline (Fig. 5g and Supplementary Fig. 10). These genes (Supplementary Fig. 10) include metalloproteinases involved in tumor invasion and metastasis (MMP1 and MMP16), transcription factors and coactivators (ZEB2, E2F5, E2F7, HNF4G, MEF2C, NR4A2, NCOA7, NCOA1, CITED2) chromatin related factors (HDAC2, TET1 and HMGN3), signaling molecules (IL7, FGF12) and relevant kinases and phosphatases (EPHA7, FYN, TNIK and DUSP6). Importantly, some of these genes are associated with super-enhancers and part of the RAS signature and about one third were also significantly downregulated by the sh5 (Supplementary Fig. 10). Changes in the expression of several of these genes observed in the RNA-seq analysis were confirmed by qPCR (Supplementary Fig. 9f).

### Top H3K36me2 methylated regions are clustered in certain genomic locations

**Figure 6:**
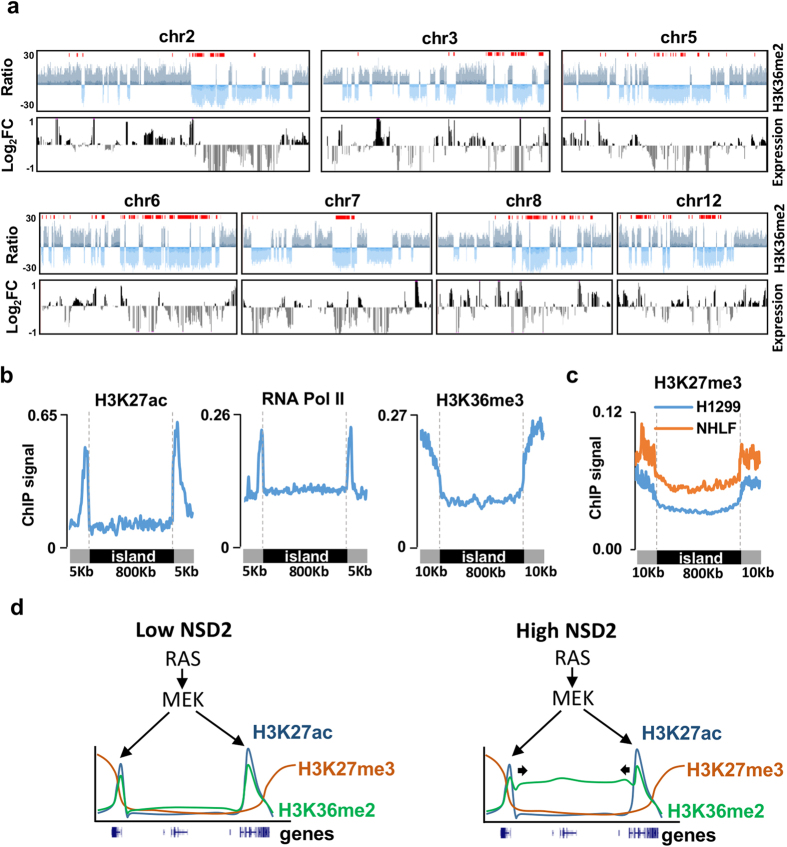
Chromosomal domains with coordinated loss of H3K36me2 and gene expression. (**a**) Smoothed H3K36me2 signal ratio (H3K36me2) and gene expression changes (FPKM) in doxycycline versus vehicle treated cells along chromosomes most enriched in top H3K36me2 islands (shown in red). Regions of retention of H3K36me2 are shown in dark blue and loss in light blue. Upregulated genes are shown in black and downregulated genes in grey. (**b**) H3K27ac and H3K36me3 levels and RNA Pol II occupancy at top H3K36me2 islands and the indicated flanking sites in H1299. (**c**) Levels of H3K27me3 at top H3K36me2 islands and flanking sites in H1299 and normal human lung fibroblasts (NHLF). (**d**) Model of NSD2 action in cell lines with RAS activating mutations. RAS signaling contributes to the establishment of super-enhancers. In the presence of low levels of NSD2, H3K36me2 is confined to enhancer regions. When levels of NSD2 are high the H3K36me2 mark spreads along regions of low H3K27me3 levels and low gene content, increasing the expression of genes located on these areas.

Interestingly, we noticed that top H3K36me2 islands were not evenly spread along the genome but clustered in certain locations, mainly on the large arms of several chromosomes (Supplementary Fig. 11). These regions are often coincident with large areas of strong H3K36me2 depletion that correlate with large domains of downregulated gene expression after NSD2 knock down (Fig. 6a). We next asked which features might be promoting the accumulation of these long domains at these particular genomic locations in cancer cells. Top H3K36me2 regions contained twice less CpG islands and 3.5 less genes per Kb than regions with low H3K36me2, suggesting that the absence of CpG islands and gene bodies might favor the spread of this mark in cancer cells that express high levels of NSD2. In accordance with this hypothesis we found that the boundaries of top H3K36me2 islands were enriched in proximal promoter regions (p-value: 4.7 × 10^−15^) and features associated with active promoters (H3K27ac, H3K36me3 and Pol II occupancy) (Fig. 6b). As expected from the negative correlation found between H3K36me2 and H3K27me3 signals, levels of H3K27me3 were low along the H3K36me2 islands compared to flanking regions (Fig. 6c). This pattern was found both in H1299 cells and normal human lung fibroblast (NHLF) that express low levels of NSD2, suggesting that low levels of H3K27me3 at certain genomic locations might facilitate the spreading of the H3K36me2 mark rather than being the consequence of the H3K36me2 spread.

## Discussion

The importance of NSD2 translocations in blood tumors has been previously demonstrated. However, its potential role in solid tumors remains less understood. As previously described, some lung cancer cell lines express high levels of NSD2 without gain of copy number. Our work suggests that in a subset of these cell lines NSD2 is likely to play a role in supporting the RAS pathway. We show that NSD2 is involved in the proliferation of several lung cancer RAS-mutant cell lines, whereas it seems of lesser importance in cells that do not have RAS mutations. Moreover, NSD2 overexpression contributes to boost E1A-Ras transformation potential. However, given the fact that NSD2 is overexpressed in a wide variety of tumors we cannot exclude that NSD2 contributes to regulate other oncogenic pathways.

NSD2 appears to contribute to the RAS pathway in H1299 cells by affecting the transcriptional output mediated by oncogenic RAS. However, while JQ1 and PD0325901 cooperate with NSD2 depletion in the downregulation of the expression of several common RAS target genes, many direct targets of NSD2 are different than those of JQ1 and PD0325901. Figure 4c exemplifies these divergent responses on RAS target genes. Genes downregulated by both JQ1 and PD0325901 (cluster 4) are enriched in super-enhancers (p < 6.32 × 10^−11^ and p < 7.4 × 10^−12^, respectively) while a significant number of genes downregulated by doxycycline (clusters 1 and 2) are located in top H3K36me2 regions (p < 4.11 × 10^−08^). Therefore, a subset of RAS target genes are marked with super-enhancers and downregulated by JQ1 or PD0325901 and a different subset have high levels of H3K36me2 and are downregulated after the NSD2 knock down.

The impact of PD0325901 in genes associated with cancer-acquired super-enhancers suggests that the RAS pathway through MEK activation controls the expression of critical transcription factors that bind to cancer-acquired super-enhancers and recruit histone acetyltransferases. Despite previous reports proposing that BRD4 plays a role in NSD2 recruitment to chromatin, we found a rather weak overlap in the transcriptional responses to JQ1 and NSD2 depletion of direct target genes (associated with cancer-acquired super-enhancers or enriched in H3K36me2, respectively) suggesting that in cancer cell lines that overexpress NSD2 its recruitment to chromatin is independent of BRD4. However, we noticed that H3K36me2 becomes enriched in H3K27ac and H3K4me1 regions after NSD2 knock down, especially at enhancer regions. In this situation residual levels of NSD2 or other members of the NSD family like NSD3 might be potentially recruited through BRD4 to acetylated regions. It is tempting to speculate that in the presence of high NSD2 levels H3K36me2 could spread from these regions into adjacent intergenic and lamina associated domains. Low gene content, decreased number of CpG islands and low levels of H3K27me3 could be facilitating the spreading of this mark at these large genomic locations, while presence of acetylated regions and active promoters might serve as boundaries. Importantly, gene density influences the portioning of the genome^39^ and enrichment of TSSs and marks of active transcription have been also found near the boundaries of topologically associating domains^40^ suggesting that higher-order physical organization of chromatin might be involved in setting the barriers to the spreading of the H3K36me2 mark. Ultimately, our results suggest that the RAS pathway through MEK activation controls functional enhancers in cancer cells and that overexpression of NSD2 causes the spread of the H3K36me2 mark from these enhancers into neighboring areas that have low levels of H3K27me3 further activating the expression of genes embedded in these regions (Fig. 6d). This particular mode of action suggests that NSD2 could also contribute to boost the transcriptional responses mediated by other oncogenic pathways responsible for the establishment or maintenance of enhancer regions in other types of cancer.

The impact of the spreading of H3K36me2 into intergenic regions caused by cancer-context overexpressed NSD2 should not be underestimated since intergenic regions constitute a critical source of regulatory complexity in mammalian cells (Nelson *et al.*)^41^. Top H3K36me2 regions contain important genes targeted by the RAS pathway. Additionally, regions chr12: 38161453-48300708 and chr8: 58830148-89198575 are both amplified and heavily marked with H3K36me2 in H1299 suggesting that the presence of this modification reinforces the expression of amplified genes. As expected from poor-gene density regions and lamina-associated domains genes marked with NSD2-dependent H3K36me2 are not as highly expressed as super-enhancer associated genes (compare Fig. 4d,e with Fig. 5g), suggesting that the spread of H3K36me2 causes a general more permissive chromatin environment around regions that otherwise have relatively low levels of transcription.

Events of long range epigenetic activation that affect the expression of genes located on genomic clusters have been previously described in prostate cancer^42^. Long domains of dysregulated gene expression that correlate with alterations in H3K4me3 levels have been described in Down’s syndrome^43^ and aberrant spreading of H3K27ac mark into long domains caused by BRD4-NUT fusions has been recently reported in NUT midline carcinoma^44^. Here we show that cancer-associated alterations in the expression the histone methyltransferase NSD2 can contribute to the formation of aberrant epigenetic domains that alter the expression of clusters of genes. Overall, long range epigenetic remodeling seems to be playing an important role in tumor progression, and perhaps other diseases, thus the dissection of the mechanisms contributing to this process appears crucial to understand the molecular basis of oncogenic transformation.

It is worth noting that NSD2 expression has been reported to be detectable by immnunohistochemistry in 27% of lung cancer biopsies but undetectable in normal tissues^10^. This makes NSD2 an attractive candidate for lung cancer therapy. While BRD4 and MEK inhibitors show redundancy in their responses through cancer-acquired super-enhancers, NSD2 inhibition affects the expression of a different subset of RAS-target genes embedded in large H3K36me2 regions and complements the effects of MEK or BRD4 inhibitors to reach a more comprehensive inhibition of oncogenic RAS-driven transcriptional programs. Therefore, combinatorial therapies using NSD2 inhibitors together with MEK or BRD4 inhibitors are likely to be effective in fighting RAS-dependent cancers with NSD2 overexpression. Additionally, NSD2 could potentially contribute to boost the transcriptional outputs of other oncogenic pathways different from RAS depending on the cancer type. Future work will aim the identification of additional tumors and cell lines that depend on NSD2 for proliferation and the oncogenic pathways involved in such dependence.

## Methods

### Cell lines and reagents

Human lung cancer cell lines (A549, NCIH1299, NCIH23, NCIH226, NCIH460, NCIH520, NCIH1703, SW1271, SW1573 and NCIH358) and blood cancer cell lines (KMS11 and RCH-ACV) were purchased from ATCC, JCRB and DSMZ. Antibodies were obtained from the following sources: NSD2 39879, H3K36me2 61019 and H3K36me2 39255 from Active Motif, NSD3 11345-1-AP from Proteintech, phospho ERK 4370 and H3K36me2 2901 from Cell Signaling, total ERK from BD Biosciences (anti-ERK1 554100 and anti-ERK2 610103), H3K36me2 ab9049 from Abcam, H3 07-690 and H3K27me3 07-449 from Merck Millipore and ACTB A5441 from Sigma Aldrich. Plasmid containing human NSD2 cDNA was purchased from Source BioScience (IMAGE ID: 100066394) and subcloned into pMSCV-FLAG-puro. The E1099K mutation was introduced using the QuikChange II XL Site-Directed Mutagenesis Kit (Agilent Technologies).

### RNA interference and establishment of stable cell lines

Doxycycline inducible pTRIPZ vectors containing different shRNAs against NSD2 and NSD3 were purchased from Dharmacon. Target sequences for NSD2 are the following; sh1 TGGAGCACACGAAGCACCA, sh2 TGTCCAGGAACGCTGAGCT, sh3 TAGAGAAAGGTGAACTTGG, sh4 TCCCGTAAGGCTTATTCAC, sh5 TATGCTCCTCTTCCTGTTC and sh6 TTCAAACTGTCCTTCTCCT. Target sequence for NSD3 is TTGGTGTAGAAATTATAAG. Lentiviral infections were performed as previously described^45^. Stable cell lines were stablish after two weeks of selection with 2 μg/ml puromycin and downregulation of NSD2 was assessed by western blot after 6 days of treatment with 0.5 μg/ml doxycycline. For clonal selection cells infected with shNT and sh3 were treated with doxycycline for 2 days and those expressing the highest levels of tRFP were sorted and plated as single cells in 96 well plates. After several weeks several clones were expanded and characterized.

### Cell extracts

To assess the expression of NSD2 and histone modifications by western blot whole cell extracts were obtained by resuspending cell pellets in SDS lysis buffer (65 mM Tris-HCl pH 7.5, 5% glycerol and 2% SDS) in the presence of proteases inhibitors. After 10 minutes in ice, lysates were sonicated, spun down and supernatants were collected. To interrogate the levels of phospho and total ERK cell pellets were resuspended in RIPA buffer (50 mM Tris-Cl pH 7.4, 150 mM NaCl, 1% NP40 and 0.25% Na-deoxycholate) supplemented with proteases and phosphatases inhibitors. After 30 minutes in ice lysates were spun down and supernatants collected.

### Proliferation curves

For proliferation curves cells were counted using a haemocytometer and plated at day 0 in triplicate for each condition, treated with 0.5 μg/ml doxycycline and/or 75 nM JQ1 and/or 10 nM PD0325901 and harvested and counted at the indicated days. In the doxycycline condition cells were pretreated with doxycycline for five days before the start of the proliferation curve to allow maximum depletion of NSD2. For colony forming assays cells were plated in triplicate and stained with crystal violet 15 days after plating. Media containing fresh doxycycline was replaced every 3–4 days. Visible colonies were counted manually.

### Cell cycle analysis

Cell pellets were fixed with 70% ethanol in PBS at 4 °C for at least one hour and stained with Hoechst 33342 in the presence of RNase A and 0.1% Triton X-100 at 4 °C overnight. Cell cycle distribution was measured using a BD LSRFortessa flow cytometer (BD Biosciences) and data analyzed using the FlowJo software.

### Xenograft experiments

Six mice were used for each experiment in which 5 × 10^6^ cells from shNT and sh3 clones were injected subcutaneously into opposite lower flanks of nude athymic mice. Doxycycline was continuously supplied in the food. Tumor diameters were measured with digital calipers three times per week and the tumor volume was calculated by the formula Volume = (width)2 × length/2. Animals were sacrificed before tumors reached a maximum volume of 3 cm3 and tumors were extracted and weighted. Mice that developed shNT tumors smaller than 0.5 cm3 at the time of sacrifice were not included in the analysis. As expected, sh3 tumors were almost undetectable in these mice. All mice procedures have been approved and are in accordance with the Spanish law (Real decreto 53/2013). Mice were maintained at the Spanish National Cancer Research Center (CNIO) under pathogen-free conditions, following the recommendations of the Federation of European Laboratory Animal Science Association (FELASA).

### RAS-mediated transformation

Phoenix ecotropic cells were transfected with pMSCV-FLAG-NSD2-Puro wild type, mutant or empty vector and virus collected and used to infect mouse embryonic fibroblasts (MEFS). Infected MEFS were selected with 2 μg/ml puromycin for one week and re-infected with viruses produced in phoenix ecotropic cells from vector pLPC-E1a-IRES-H-ras V12 as previously described^46^. Three days later 100,000 cells were plated in 10 cm dishes and stained with crystal violet after 20 days. Foci number and size were determined using ImageJ^47^.

### Analysis of histone modifications

For preparation of the total histones, cells in culture were harvested with trypsin, washed with cold PBS, resuspended in 0.5% Triton X-100 (v/v), incubated for 20 min on ice, and spun for 10 min at 2000 rpm in a bench-top centrifuge to collect nuclear fraction as a pellet, which was resuspended in a small volume of 0.4N HCl and incubated overnight at 4C to extract histones into soluble fraction. Insoluble material was removed by centrifugation. For processing histone samples for mass spectrometry analysis, variations of published methods were employed^48^,^49^. Briefly, 5 μg of the acid extracted histones were lyophilized, dissolved in 30 μl of PBS, and incubated with 20 μl of acetylation reagent (20 μl of acetic anhydride in 3 ml of acetonitrile) for 2 hr at room temperature to acetylate un-modified and mono-methylated lysine residues, before digestion with trypsin which would cleave only after Arg residues with all Lys residues modified either *in vivo* or *in vitro*. One fifth volume of the acetylated histones was incubated with 0.02 μg of MS-grade trypsin (Pierce) overnight at 37 °C in a total volume of 100 μl adjusted with 100 mM ammonium bicarbonate. Digested peptides were lyophilized, dissolved in 30 μl PBS, and incubated with 20 μl of N-terminal modification reagent (20 μl of phenyl isocyanate in 3 ml of acetonitrile) for 2 hr at room temperature, resulting in phenyl isocyanation of the N-termini of the tryptic peptides. Samples were lyophilized to remove acetonitrile, dissolved in 50 μl of 0.1% formic acid, before LC/MS analysis using Dionext Ultimate 3000 UHPLC system coupled in line with Thermo QExactive Orbitrap high resolution mass spectrometer. Identification of the individual peptides with different methylations was done by analysis of the MS2 spectra and by co-migration with isotopically labeled standard peptides. Peak areas of the extracted ion chromatogram for four major isotopes in primary MS as a percentage of total peak areas of all peptides with the same amino acid sequence was used as a representation of the abundance for a particular modification status.

Levels of H3K36me2 were also analyzed using the AlphaLISA dimethylated-Histone H3 Lysine 36 (H3K36me2) Cellular Detection Kit (Perkin Elmer) following the manufactures protocol.

### RNA-seq

Cells were trypsinized at day 7 of the growth curve and total RNA was extracted using the RNeasy kit (Qiagen). Two biological replicates were used for condition. From 1 μg of total RNA PolyA+ fraction was purified and randomly fragmented, converted to double stranded cDNA and processed through subsequent enzymatic treatments of end-repair, dA-tailing, and ligation to adapters as in Illumina’s “TruSeq Stranded mRNA Sample Preparation Part # 15031047 Rev. D” kit. Adapter-ligated library was completed by PCR with Illumina PE primers (8 cycles). The resulting purified cDNA library was applied to an Illumina flow cell for cluster generation and sequenced for 50 bases in a single-read format (Illumina HiSeq 2000). Reads were quality-checked with FastQC (http://www.bioinformatics.babraham.ac.uk/projects/fastqc/) and aligned to the human genome (GRCh37/hg19) with TopHat-2.0.10^50^, using Bowtie 1.0.0^51^ and Samtools 0.1.19^52^, allowing two mismatches and five multihits. Transcripts assembly, estimation of their abundances and differential expression were calculated with Cufflinks 2.2.1^50^, using the human genome annotation data set GRCh37/hg19 from the UCSC Genome Browser. Transcripts with FPKM expression values lower than 0.05 in both conditions were considered not expressed and were excluded from further analysis. Heatmaps were generated using GENE-E (http://www.broadinstitute.org/cancer/software/GENE-E) and box plots using BoxPlotR (http://boxplot.tyerslab.com/).

### ChIP-seq

Chromatin immunoprecipitation (ChIP) assays were performed according to the Millipore protocol. Cells were fixed using 1% formaldehyde, harvested, resuspended in ChIP lysis buffer (1% SDS, 10 mM EDTA, 50 mM Tris-HCl, pH 8.1) and sonicated using Bioruptor Pico (Diagenode) to generate fragments of 150 to 300 bp. Soluble chromatin was diluted 8 fold in ChIP RIPA buffer (10 mMTris–HCl, pH 7.5, 140 mMNaCl, 1 mMEDTA, 0.5 mMEGTA,1%Triton X-100, 0.1% SDS, 0.1% Na-deoxycholate) and incubated with Dynabeads Protein A (Invitrogen) coupled to H3K36me2 antibody ab9049 from Abcam. After incubation, the immunocomplexes were washed sequentially with Low Salt Wash Buffer (0.1% SDS, 1% Triton X-100, 2 mM EDTA, 20 mM Tris-HCl, pH 8.1, 150 mM NaCl), High Salt Wash Buffer (0.1% SDS, 1% Triton X-100, 2 mM EDTA, 20 mM Tris-HCl, pH 8.1, 500 mM NaCl), LiCl Wash Buffer (0.25 M LiCl, 1% NP40, 1% deoxycholate, 1 mM EDTA, 10 mM Tris-HCl, pH 8.1) and TE. Immunocomplexes were eluted in ChIP elution buffer (1%SDS, 0.1 M NaHCO3) and the crosslinking was reverted overnight at 65 °C. Samples were treated with Proteinase K and RNase A and DNA was extracted using the QIAGEN PCR purification kit. Purified chromatin was used for library construction in the ChIP-seq experiments or for qPCR amplification.

### Library Generation and Sequencing

Inmmunoprecipitated DNA (15 ng) for each condition and the pooled inputs were used for library construction. Briefly, samples were processed through subsequent enzymatic treatments of end-repair, dA-tailing, and ligation to adapters with “NEXTflex ChIP Sequencing kit” from Bioo Scientific (part # 5143). Adapter-ligated libraries were completed by limited-cycle PCR (13 cycles) and extracted with a double double-sided SPRI size selection. Average fragment size is 450 bp, 380 bp for the pool inputs, from which 120 bp correspond to adaptor sequences. Libraries were applied to an Illumina flow cell for cluster generation and sequenced on an Illumina HiSeq 2000 by following manufacturer's protocols.

### ChIP-Seq Data analysis

Sequence analysis was carried out using Galaxy (https://main.g2.bx.psu.edu/) and Galaxy Cistrome (http://cistrome.org/). Three biological ChIP replicates for H3K36me2 were mapped to the human genome build hg19 using Bowtie^51^ and pooled, resulting in about 140 × 10^6^ uniquely mapped reads for each treatment. Bigwig files were generated and displayed in the UCSC Genome Browser. Genomic intervals marked by H3K36me2 were determined using SICER^53^ (window size, 200; fragment size, 260; gap size, 600; FDR, 0.01) and top H3K36me2 islands were identified using ROSE (https://bitbucket.org/young_computation/rose)^32^,^35^. Briefly, H3K36me2 intervals were stitched together if they were within 12.5 kb, and ranked by their H3K36me2 signal. Densities of H3K36me2 H3K36me3, H3K27ac and RNA Pol II signals at given genomic features and metagene representations were calculated using bamToGFF (https://github.com/bradnerComputation/pipeline/blob/master/bamToGFF.py). For visualization of the changes in H3K36me2 levels BAM files with aligned reads generated using RUbioSeq3.7^54^ were converted to sorted BED files. Reads per nucleotide were counted for each sample with BEDtools 2.23.0^55^ and divided by the total number of reads in the sample. Reads counts were subtracted from the pair of compared samples (doxycycline minus vehicle) and finally stored in BigWig files to allow visualization in the UCSC Genome Browser.

### Gene set enrichment analysis (GSEA)

For GSEAPreranked^28^ genes were pre-ranked according to the statistic test of fold change for each treatment obtained in the RNA-seq analysis, setting ‘gene set’ as the permutation method and with 1000 permutations. Gene sets from the Molecular Signature Database (MSigDB) KRAS.300_UP.V1_UP, KRAS.600_UP.V1_UP, KRAS.50_UP.V1_UP and KRAS.KIDNEY_UP.V1_UP, all upregulated by Ras activation were significantly enriched in our experiments. Comparison of the of RAS signatures between treatments was done as previously described^33^. Briefly, genes included in above signatures were compiled to elaborate a Ras signature gene set that was used to select genes that were significantly downregulated by doxycycline, JQ1 or PD0325901 treatments (205 genes at FDR < 0.05).

### Super-enhancer analysis

SICER was used to identify H3K27ac intervals in H1299 and normal lung and super-enhancers were identified using ROSE. Briefly, H3K27Ac intervals were stitched together if they were within 12.5 kb, and ranked by their H3K27Ac signal. We identified super-enhancers acquired in lung cancer cells (H1299) relative to normal lung as previously suggested for other cell types^56^. Briefly, super-enhancers intervals in H1299 and normal lung were concatenated and merged and the density of H3K27ac at these combined intervals in each sample was calculated. Regions with a ratio of H3K27ac signal in H1299 vs. normal lung higher than 10 fold were considered cancer-acquired super-enhancers while regions with a ratio between 2 and 0.5 were considered shared super-enhancers.

### Mapping of elements to genes

Super-enhancers were mapped to the nearest gene using GREAT^36^. This analysis considers 17,744 curated TSSs from canonical isoforms of UCSC Known Genes. Same set of genes were used to identify genes with RNA PolII in −/+5 Kb around the TSS, regulatory regions (−/+5 Kb around the TSS) that overlap with top H3K36me2 islands and coding regions with or without H3K36me3.

### qPCR

For the analysis of ChIP samples the immunoprecipitated chromatin and the input were directly used for qPCR. For analysis of mRNA levels total RNA was reverse transcribed. Real-time PCR analysis was run using Power SYBR Green PCR Master mix (Applied Biosystem). Specific oligonucleotides for amplification are listed in Supplementary Table 5.

### Statistical Methods

The significance of differences in gene expression or signal density between specific categories was analyzed using paired Student’s t test. The significance of differences in cell number, colony count and tumor size and weight was analyzed using Student’s t test. The significance of the number of genes overlapping between treatments was calculated using the hypergeometric test. P-values are either shown or labeled as the following *p-value < 0.05, **p-value < 0.005 and ***p-value < 0.0005.

### Source of public data

Data on gene expression and copy number in lung tumors was generated by The Cancer Genome Atlas (TCGA) and normalized data was downloaded from the UCSC Cancer Browser. Normalized data on gene expression and copy number in lung cancer cell lines was downloaded from The Cancer Cell Line Encyclopedia (CCLE) website (http://www.broadinstitute.org/ccle/home). ChIP-seq for H3K27ac (DRX015250), RNA Pol II (DRX015243), H3K4me1 (DRX015247), H3K27me3 (DRX015249) and H3K36me3 (DRX015248) in H1299 were previously published^57^ and raw data was downloaded from the DNA Data Bank of Japan. Raw data for H3K27ac in normal human lung (GSM1013123) was generated by the Roadmap Epigenomics Project and downloaded from GEO. Data for H3K27me3 in NHLF was generated by ENCODE at the Bernstein laboratory (GSM733764). Lamina associated domains (LADs) in Tig3 cells were previously described^58^ and downloaded from the UCSC Genome Browser tracks.

The RNA-seq and ChIP-seq data has been deposited in the GEO repository (accession number GSE73696)

## Additional Information

**How to cite this article**: García-Carpizo, V. *et al.* NSD2 contributes to oncogenic RAS-driven transcription in lung cancer cells through long-range epigenetic activation. *Sci. Rep.* **6**, 32952; doi: 10.1038/srep32952 (2016).

## Supplementary Material

Supplementary Information

Supplementary Table 1

Supplementary Table 3

Supplementary Table 4

Supplementary Table 5
