# Supplementary material for: NSD2 contributes to oncogenic RAS-driven transcription in lung cancer cells through long-range epigenetic activation
Source: Sci Rep. 2016 Sep 8;6:32952. doi: 10.1038/srep32952 (PMC5015087; doi:10.1038/srep32952)
Supplement: Supplementary Information [file srep32952-s1.pdf]

SUPPLEMENTARY INFORMATION

NSD2 supports oncogenic RAS-driven transcription in lung cancer cells through long-range epigenetic activation.

Verónica García-Carpizo, Jacinto Sarmentero, Bomie Han, Osvaldo Graña, Sergio Ruiz-Llorente, David G. Pisano, Manuel Serrano, Harold B. Brooks, Robert M. Campbell and Maria J. Barrero

SUPPLEMENTARY FIGURE LEGENDS

Supplementary Figure 1. Differential expression of histone lysine methyltransferases between normal and cancer lung tissues.

(**a**) Box plot of mRNA levels of 24 histone lysine methyltransferases in paired normal (white) and tumor (pink) tissues analyzed by RNA-seq by The Cancer Genome Atlas (TCGA) in lung adenocarcinoma and squamous cell carcinoma patients. (**b**) P-values regarding the differences in expression for each methyltransferase between normal and tumor tissue of adenocarcinoma (AD) and squamous cell carcinoma (SCC) patients calculated using paired *t*-test are shown.

Supplementary Figure 2. NSD2 expression in cancer cell lines and shRNA-mediated knock down.

(**a**) Correlation between expression levels and copy number (CN) in lung cancer cell lines according to data from The Cancer Cell Line Encyclopedia (CCLE). (**b**) Representation of NSD2 described variants and domains. The position of the different shRNA used in this study is shown. (**c**) Levels of NSD2 determined by western-blot of H1299 cells transduced with a non target shRNA (shNT) and six different shRNAs against NSD2 and treated with vehicle (-) or doxycycline (+) for 6 days. (**d**) Growth curves (left) and levels of NSD2 determined by western blot (right) of KMS11 cells transduced with a doxycycline inducible non target shRNA (shNT) and two different shRNAs against NSD2 (sh3 and sh5) in the presence of vehicle (DMSO) and doxycycline. **p-value<0.005 and ***p-value<0.0005 determined by *t*-test. (**e**) Growth curves (left) and levels of NSD2 determined by western blot (right) of RCH-ACV cells transduced with a doxycycline inducible non target shRNA (shNT) and two different shRNAs against NSD2 (sh3 and sh5) in the presence of vehicle (DMSO) and doxycycline. **p-value<0.005 and ***p-value<0.0005 determined by t-test.

Supplementary Figure 3. Knock down of NSD3 has no effect on the proliferation of H1299 cells.

(a) Levels of NSD2 and NSD3 determined by western-blot in H1299 expressing non target shRNA (Control), knock down for NSD2 (NSD2 KD), knock down for NSD3 (NSD3 KD) and knock down for

both NSD2 and NSD3 (Double KD). **(b)** Growth curves of the corresponding cell lines. Graph shows mean and standard deviation of biological triplicates from one representative experiment out of three. *p-value<0.05 by *t*-test.

Supplementary Figure 4. Isolation and characterization of clonal populations.

(**a**) Levels of NSD2 determined by western-blot of clonal populations of one clone of H1299 cells transduced with a non target shRNA (clone C9) and two different clones of H1299 transduced with sh3 against NSD2 (clones A9 and A3) treated with vehicle (-) or doxycycline (+) for 6 days. (**b**) Growth curves of clones described in A in treated with vehicle or doxycycline. Graph shows mean and standard deviation of three replicates from one representative experiment out of two. **p-value<0.005 and ***p-value<0.0005 determined by *t*-test. (**c**) Colony forming assays of cell lines described in A in the presence of vehicle (-) or doxycycline (+).Upper panel shows crystal violet staining of colonies. Graph shows mean and standard deviation of colony count in three replicates from one representative experiment out of four. *p-value<0.05 and **p-value<0.005 determined by t-test. (**d**) Cell cycle profile of clones A9 and A3 treated with vehicle or doxycycline determined by Hoechst. The mean and standard deviation of the percentage of cells found in G0/G1 in three biological replicates are depicted. Differences between treatments were found significant in both clones by t-test (p<0.05).

Supplementary Figure 5. Lung cancer cell lines without RAS mutations are insensitive to the NSD2 knock down and the NSD2 knock down cooperates with JQ1 treatment.

(a) Levels of NSD2 determined by western-blot of H1703 cells transduced with sh3 shRNA against NSD2 treated with vehicle or doxycycline for 6 days. (**b**) Colony forming assays of cell lines described in a in the presence of vehicle or doxycycline. Left panel shows crystal violet staining of colonies. Graph shows mean and standard deviation of colony count in three biological replicates. **(c)** Levels of NSD2 determined by western-blot of H520 cells transduced with sh3 shRNA against NSD2 treated with vehicle or doxycycline for 6 days. (**d**) Colony forming assays of cell lines described in c in the presence of vehicle or doxycycline. Left panel shows crystal violet staining of colonies. Graph shows mean and standard deviation of colony count in three biological replicates.

(**e**) Growth curves of H1299 transduced with doxycycline inducible non target shRNA (shNT) and two different shRNAs against NSD2 (sh3 and sh5) in the presence of vehicle (DMSO), doxycycline and/or 75 nM BRD4 inhibitor JQ1. Graph shows mean and standard deviation of three replicates from one representative experiment out of two. **p-value<0.005 and ***p-value<0.0005 determined by *t*-test comparing each treatment to vehicle.

Supplementary Figure 6. RAS signature is downregulated by the three treatments.

(a) mRNA levels of *MYC* after the indicated treatments. *p-value<0.05 by *t*-test. (**b**) Overlap of genes found in the leading edge analysis of gene sets upregulated by KRAS that are significantly down regulated by the three independent treatments. (**c**) Box plot representation of genes induced by KRAS and commonly downregulated by the three independent treatments shown in B shows cooperativity on combinatory treatments. (**d**) Levels of phosphor ERK and total ERK after the indicated treatments in the H1299 sh3 line determined by western blot.

Supplementary Figure 7. Super-enhancer analysis in H1299 and normal lung.

(**a**) Distribution H3K27Ac signal at enhancers in lung cancer cell line H1299 and normal human lung tissue used to identify super-enhancers. (**b**) Left panel shows the ratio of H3K27ac in H1299 versus normal lung at merged super-enhancers identified in the two samples. Super-enhancers with a ratio higher than 10 were considered to be cancer-acquired. Super-enhancers with a ratio between 2 and 0.5 were considered to be shared between H1299 and normal lung. Right panel shows gene tracks of H3K27ac at four different genes found to have super-enhancers in H1299 and not in normal lung. (**c**) Box plot of mRNA levels of genes associated with cancer-acquired enhancers in paired normal (N) and tumor (T) tissues analyzed by RNA-seq by The Cancer Genome Atlas (TCGA) in lung adenocarcinoma (LAD; 57 samples) and squamous cell carcinoma (SCC; 51 samples) patients. P-values were calculated using paired *t*-test.

Supplementary Figure 8. Profiles of the H3K36me2 signal in H1299.

(**a**) Correlation coefficient values of the H3K36me2 signal in vehicle or doxycycline H1299 treated cells, several histone modifications and RNA Pol II signal. (**b**) Average density of H3K36me2 signal

in H1299 cells around the transcription start site (TSS) genes that are expressed (ON) or not expressed (OFF) according to presence of RNA Pol II on the TSS. X-axis corresponds to Kb. (**c**) Average density of H3K36me2 signal around CpG islands. X-axis corresponds to Kb. (**d**) Metagene representation of average density of H3K36me2 and H3K36me3 at genes that are marked with H3K36me3 at the coding region. The x axis shows the start and end of the coding region flanked by 5 Kb upstream of the start.

Supplementary Figure 9. Validation of the H3K36me2 signal and gene expression changes in H1299.

(**a**) Gene tracks of H3K27ac, H3K36me3 and H3K36me2 in H1299 around the *HMGA2* gene. Intervals considered super-enhancers (SE) are also shown. Interval corresponding to top H3K36me2 signal is shown in red. (**b**) ChIP coupled to qPCR carried out with two different antibodies against H3K36me2 (Active Motif 61019 in blue and Abcam ab9049 in Orange) show H3K36me2 levels around the *HMGA2* gene. Coordinates show distance from the *HMGA2* TSS in Kb. Levels were normalized to the input and plotted relative to the maximum enrichment as 1. (**c**) Same as in **a,** but showing a genomic region around the *MYC* gene. (**d**) ChIP coupled to qPCR carried out as in **b** shows H3K36me2 levels around the *MYC* gene. Coordinates indicated distance from the *MYC* TSS. Data was normalized to input (**e**) ChIP coupled to qPCR interrogating the levels of H3K36me2 (Active Motif 61019) at different genomic locations around the *HDAC2* and *MYC* genes in the presence of vehicle or doxycycline. Coordinates indicate distance from the corresponding gene *TSS*. Loss of H3K36me2 after the NSD2 knock down is appreciated at intergenic regions downstream of the *HDAC2* gene, while enrichment is observed at the *MYC* super-enhancer (amplicon G). Data was normalized to input. (**f**) qPCR validation of the expression of several NSD2 target genes after different treatments. Levels were normalized to housekeeping gene *HPRT*.

Supplementary Figure 10. Expression patterns of genes in the leading edge of the H3K36me2 signature.

Clustered heat map of the expression of leading edge genes contributing the most to the H3K36me2 signature (Fig. 5f). The presence of super-enhancers in H1299, the overlap with the RAS signature and downregulation by the sh5 are indicated in orange, blue and green respectively.

Supplementary Figure 11. Genome graphs showing changes in H3K36me2 levels.

Chromosome ideograms showing smoothed H3K36me2 ratio of doxycycline minus vehicle treated cells. Positive values in dark blue depict H3K36me2 retention and negative values in light blue depict loss of H3K36me2 after NSD2 knock down. Red lines show top H3K36me2 islands in vehicle treated cells. Green squares highlight the regions with most dramatic loss of H3K36me2.

Supplementary Table 1. Genes downregulated in H1299 cells transduced with sh3 after doxycycline, JQ1 and PD0325901 treatments

Overlap with RAS and super-enhancer signatures and top H3K36me2 islands is also indicated.

Supplementary Table 2. Enrichment of gene expression changes in relevant signatures.

Gene set enrichment analysis (GSEA) of changes in gene expression caused by independent and combined treatments showing normalized enrichment scores (NES), p-values and false discovery rate (FDR) for the indicated KRAS upregulated gene signatures (from the Molecular Signature Database) and the super-enhancer and H3K36me2 signatures. All treatments were compared to vehicle (DMSO) treatment. NS corresponds to not significant.

Supplementary Table 3. Genes included in the signatures used in this study

Supplementary Table 4. TOP H3K36me2 islands found in H1299 cells

Densities of H3K36me2 signal in vehicle and doxycycline treated cells and genes overlapping (-/+5kb around the TSS) with each island are also shown.

Supplementary Table 5. Oligonucleotides used in this study.

Supplementary Figure 1

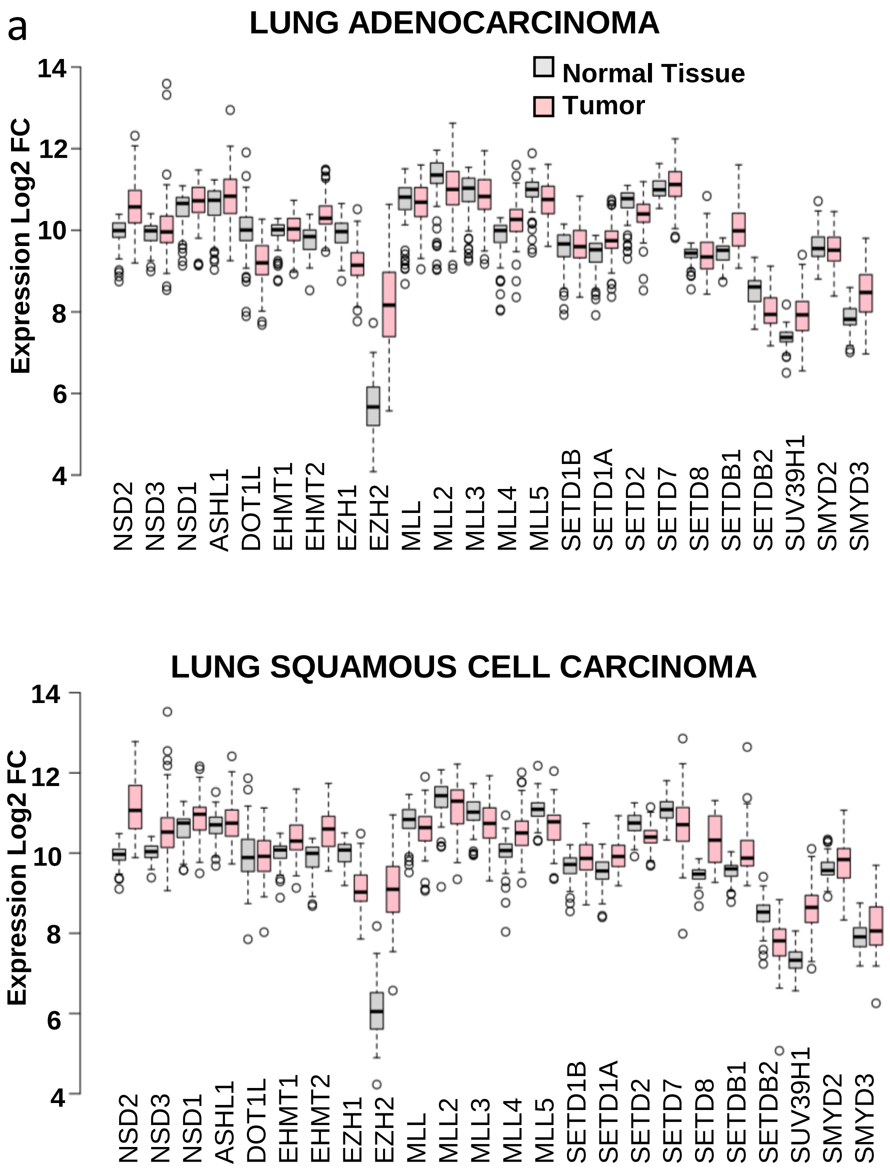

b

Gene	AD	SCC
EZH2	8.87E-20	9.55E-27
NSD2	2.86E-09	1.7E-15
EZH1	1.42E-15	5.51E-13
EHMT2	2.88E-11	6.62E-10
MLL4	5.67E-06	1.24E-07
SETD2	6.86E-05	4.7E-06
EHMT1	0.223974	5.67E-06
NSD3	0.127536	1.02E-05
SETD8	0.85279	0.00673
MLL3	0.719892	0.009474
NSD1	0.047409	0.010969
SMYD3	4.39E-07	0.013786
SETDB2	3.22E-09	0.013797
SUV39H1	5.3E-09	0.018986
SETD1A	0.000239	0.063851
MLL2	0.135926	0.075454
SMYD2	0.454255	0.117627
SETD1B	0.401735	0.130288
ASHL1	0.004955	0.164597
SETDB1	7.48E-11	0.176886
SETD7	0.232356	0.309182
MLL5	0.023451	0.432175
MLL	0.653042	0.440098
DOT1L	4.53E-09	0.884337

Supplementary Figure 2

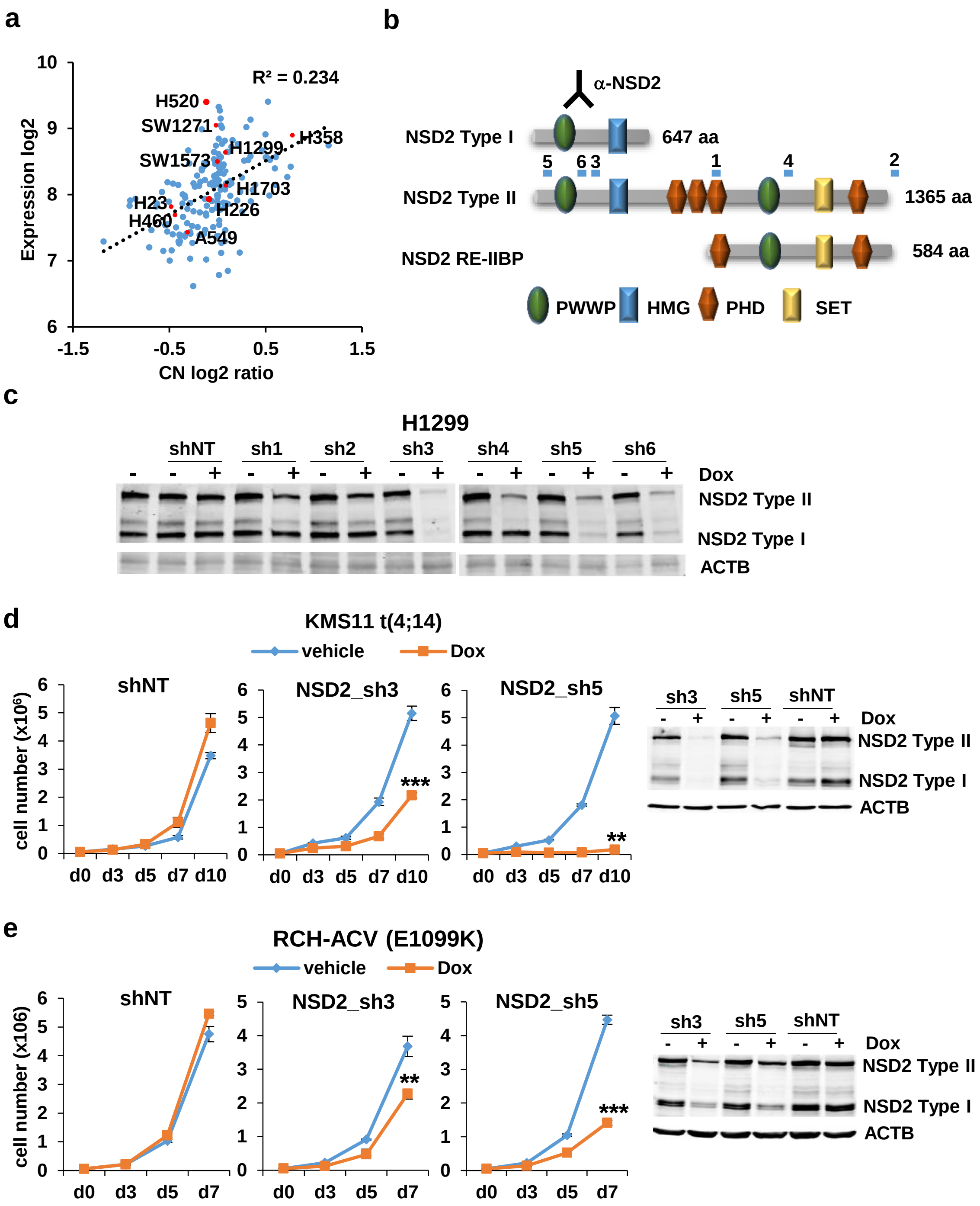

Supplementary Figure 3

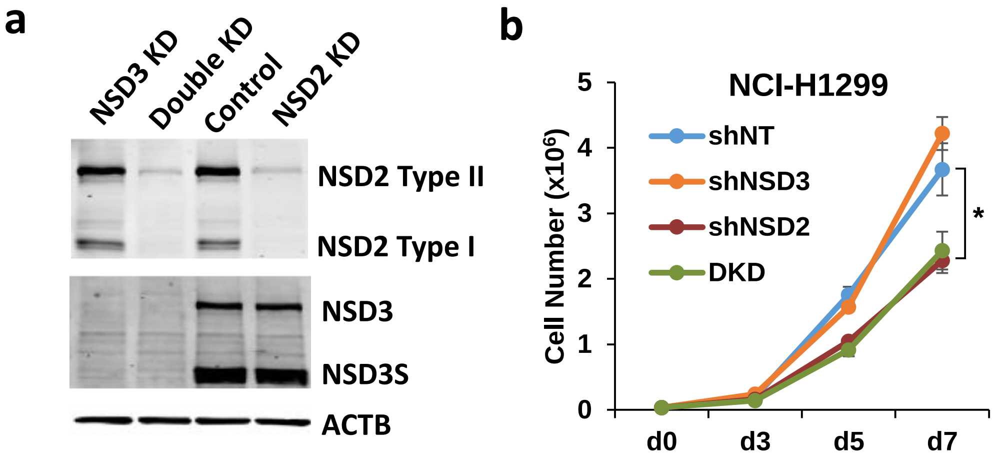

Supplementary Figure 4

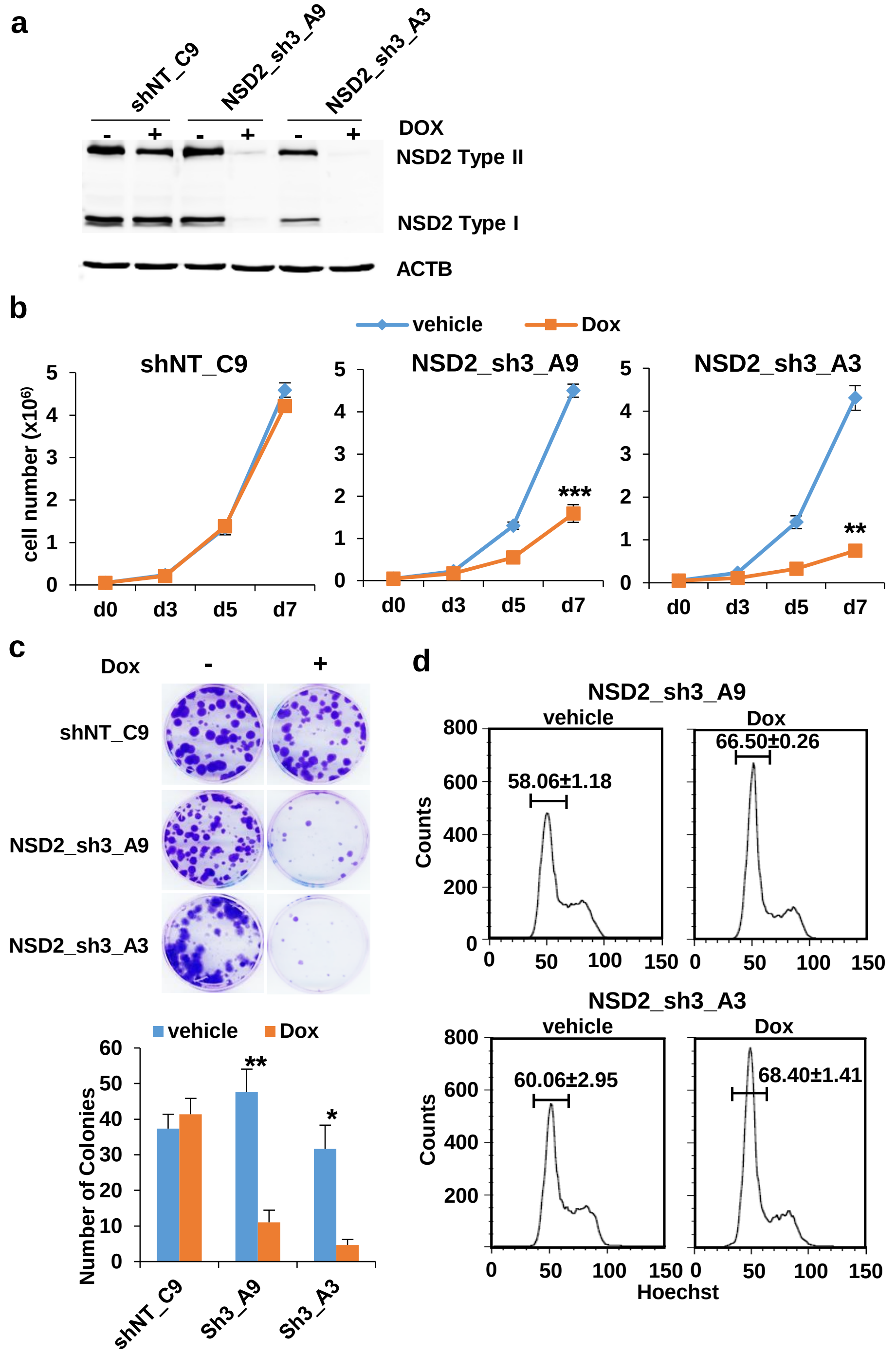

Supplementary Figure 5

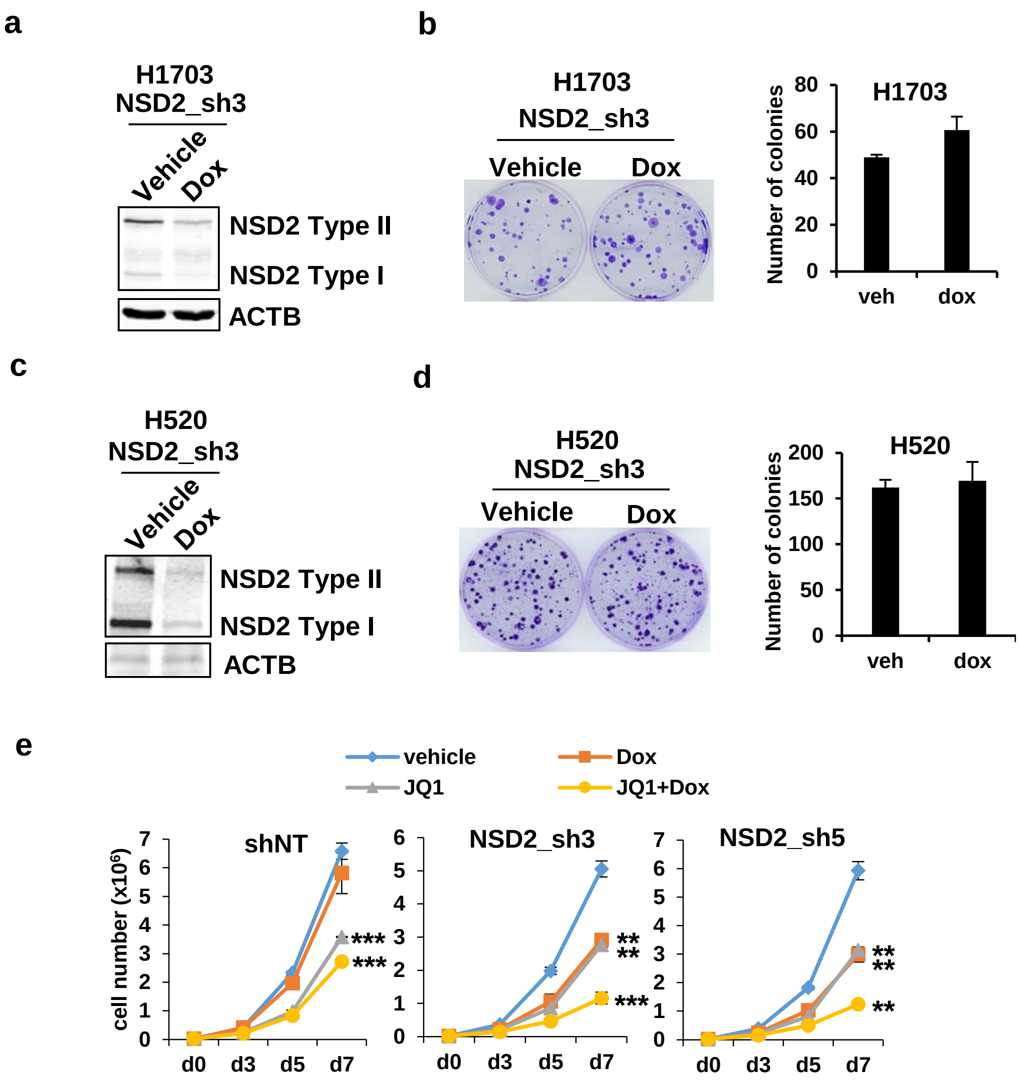

Supplementary Figure 6

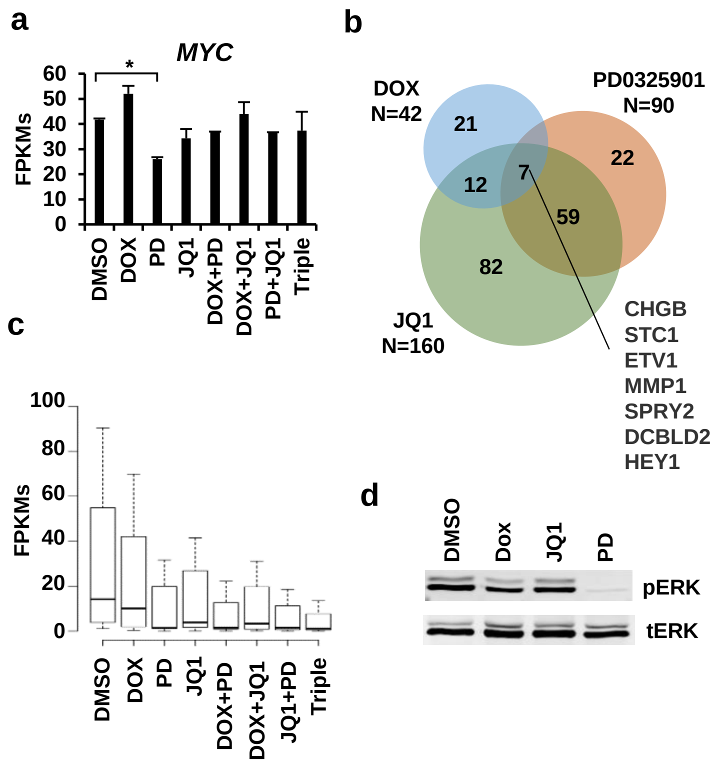

Supplementary Figure 7

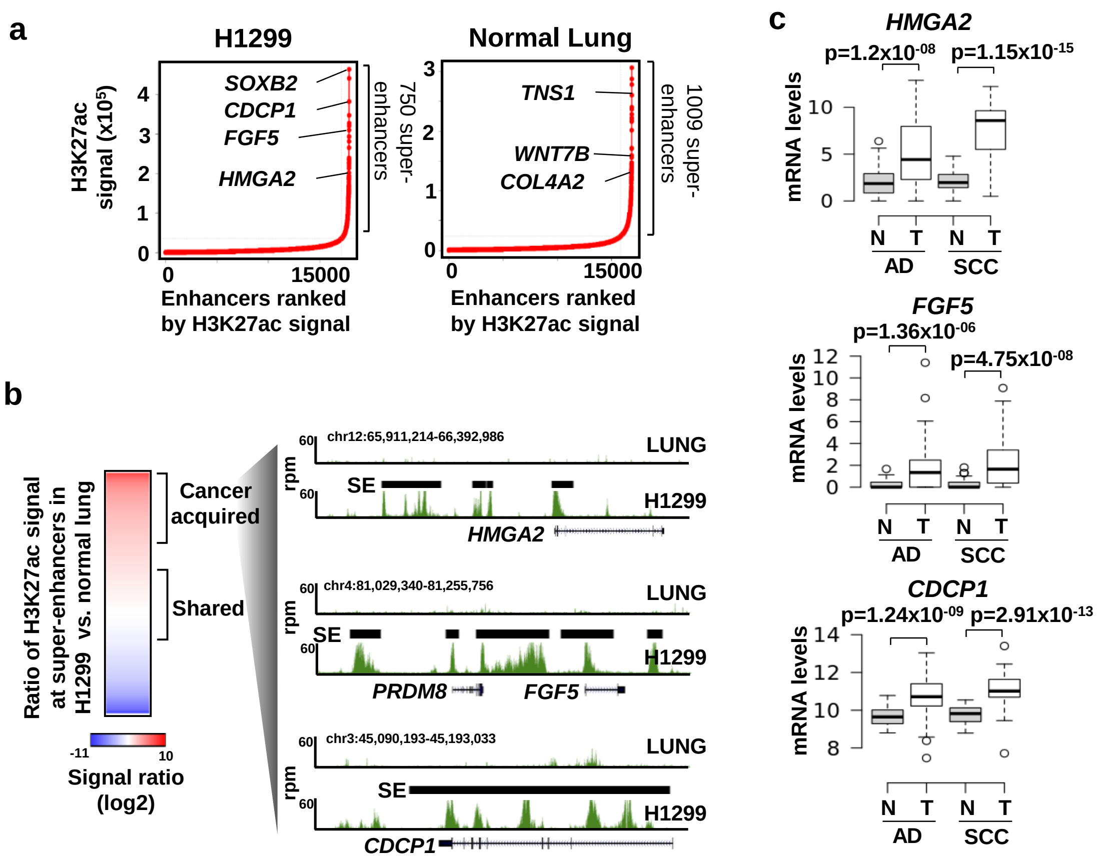

Supplementary Figure 8

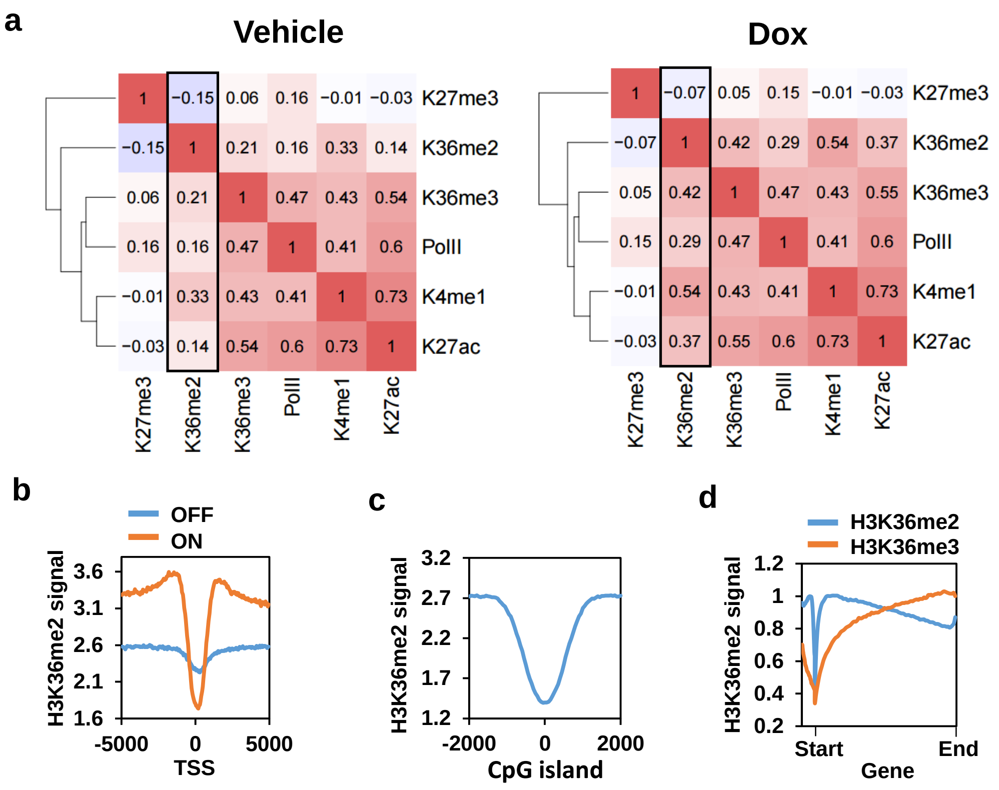

Supplementary Figure 9

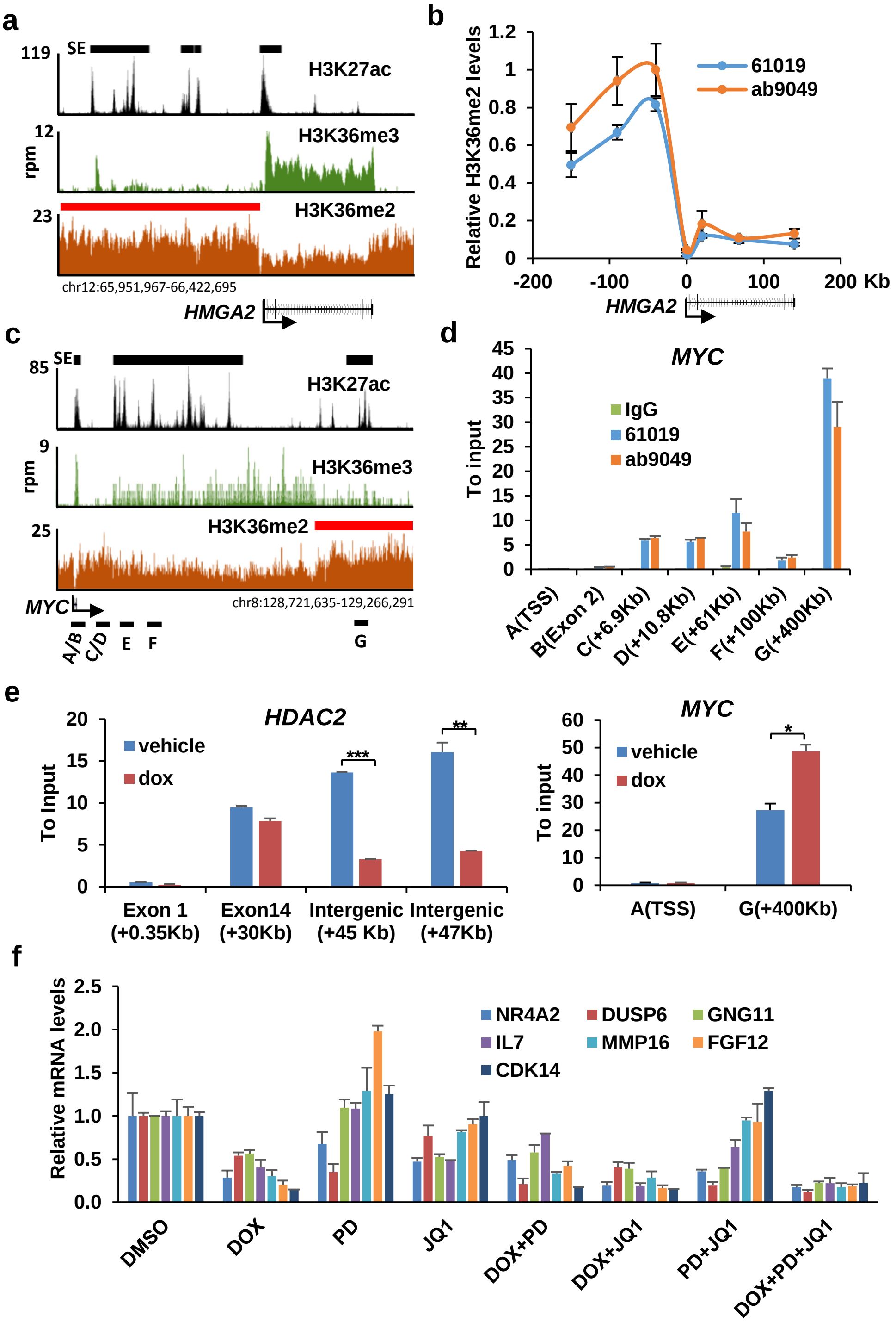

Supplementary Figure 10

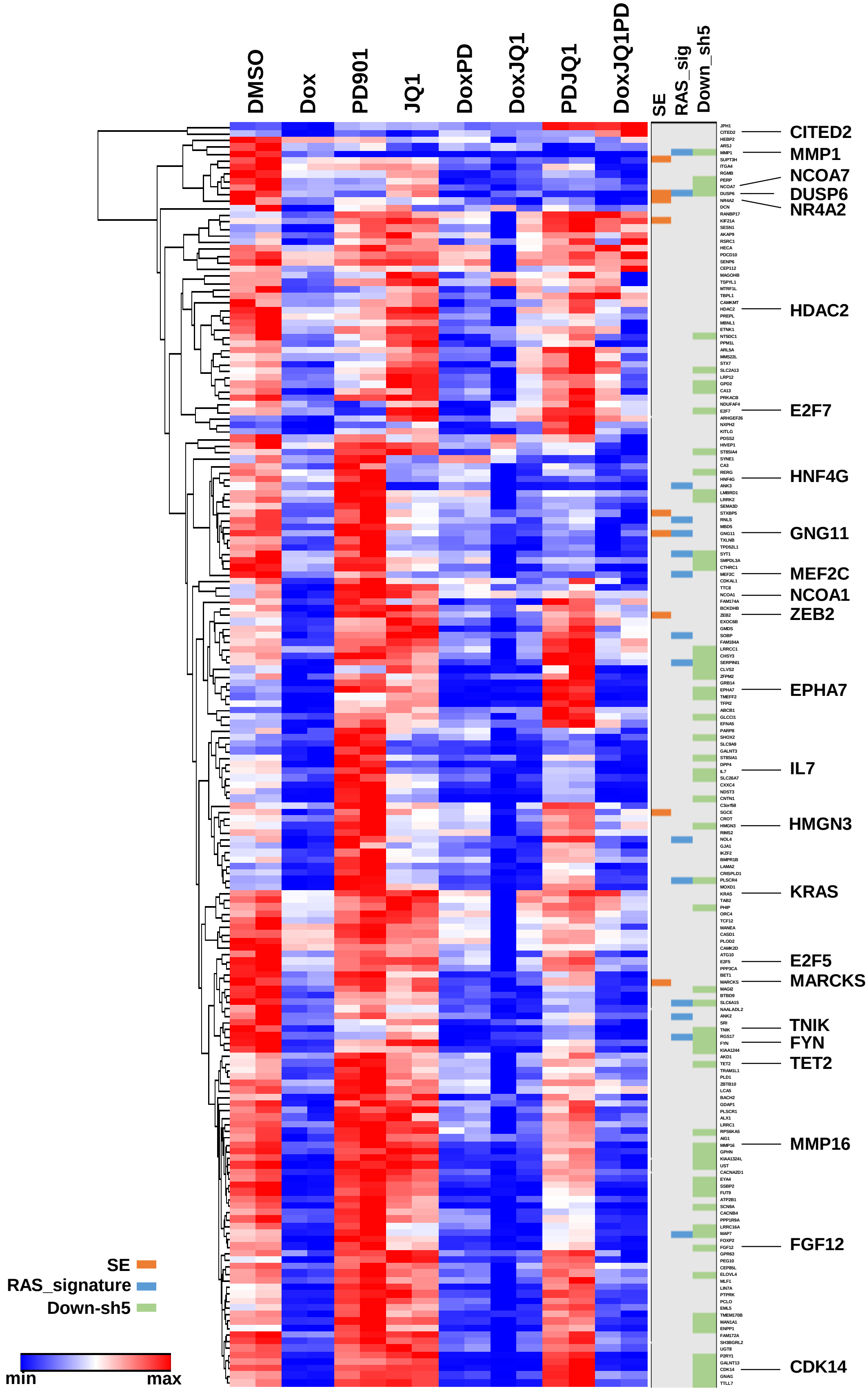

Supplementary Figure 11

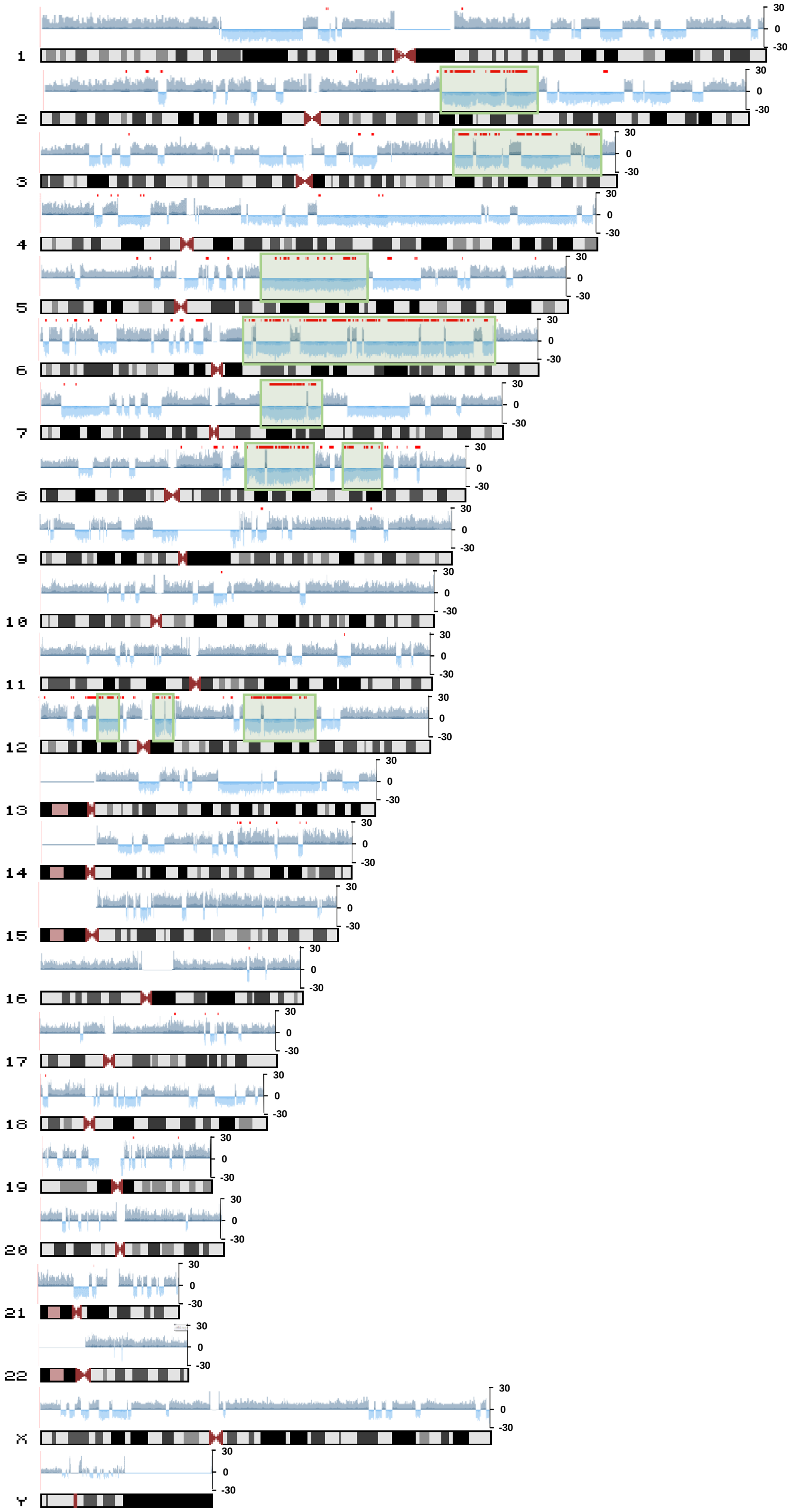

Supplementary Table 2

	Gene Set	KRAS.300_UP.V1_UP	KRAS.600_UP.V1_UP	KRAS.50_UP.V1_UP	KRAS.KIDNEY_UP.V1_UP	Cancer-acquired SE	Shared_SE	H3K36me2_signature
DOX	NES	-1.36	NS	-1.22	-2.25	NS	NS	-3.10
	p-value	0.044	NS	0.138	0	NS	NS	0
	FDR	0.137	NS	0.27	< 0.001	NS	NS	< 0.001
JQ1	NES	-2.2	-2.4	-1.75	-1.8	-2.51	-1.21	NS
	p-value	0	0	0.006	0	0	0.041	NS
	FDR	< 0.001	< 0.001	0.004	0.002	< 0.001	0.129	NS
PD	NES	-2.12	-2.27	-2.12	NS	-2.49	-1.60	NS
	p-value	0	0	0	NS	0	0	NS
	FDR	< 0.001	< 0.001	< 0.001	NS	< 0.001	0.011	NS
DOX+JQ1	NES	-1.94	-2.13	-1.77	-2.36	-1.89	-1.07	-2.50
	p-value	0	0	0.005	0	0	0.278	0
	FDR	< 0.001	< 0.001	0.004	< 0.001	0.001	0.313	< 0.001
DOX+PD	NES	-2.11	-2.31	-2.03	-2.24	-1.96	-1.06	-2.78
	p-value	0	0	0	0	0	0.197	0
	FDR	0.001	< 0.001	< 0.001	< 0.001	< 0.001	0.304	< 0.001
JQ1+PD	NES	-2.31	-2.46	-2.11	-1.94	-2.49	-1.52	NS
	p-value	0	0	0	0	0	0	NS
	FDR	< 0.001	< 0.001	< 0.001	< 0.001	< 0.001	0.008	NS
Triple	NES	-2.3	-2.47	-2.15	-2.5	-2.36	-1.21	-2.45
	p-value	0	0	0	0	0	0.054	0
	FDR	< 0.001	< 0.001	< 0.001	< 0.001	< 0.001	0.117	< 0.001
